# Precision hyperbaric oxygen therapy: a biomarker-driven framework

**DOI:** 10.1186/s12967-026-08722-w

**Published:** 2026-07-30

**Authors:** Halimureti Simayijiang, Yueling Lin, Tingting Zhong, Zhuo Li, Kun Zhang, Ying Long

**Affiliations:** 1 South China Hospital, Medical School, Shenzhen University, Shenzhen, P.R. China; 2 Yiyuan Longevity Technology (Shenzhen) Co., Ltd, Shenzhen, P.R. China; 3 Department of Hyperbaric Oxygen, Shenzhen People’s Hospital, Shenzhen, P.R. China

**Keywords:** Hyperbaric oxygen therapy, Precision medicine, Biomarkers, Pressure–time integral (PTI), Cumulative oxygen exposure, Composite Response Index (CRI)

## Abstract

**Background:**

Hyperbaric oxygen therapy (HBOT) remains limited by insufficient individualization, a lack of biomarker-guided dosing, and heterogeneous clinical outcomes across indications and patient populations. We propose a translational, biomarker-driven framework for treating HBOT as a measurable and titratable intervention. This narrative review presents a conceptual framework rather than a formal systematic review or a clinically validated dosing algorithm.

**Methods:**

Dosing is quantified using pressure–time integral (PTI) and cumulative oxygen exposure. A three-tier biomarker architecture supports decision-making: proximal sensors (redox balance, mitochondrial function, endothelial and microcirculatory responses), mechanistic mediators (inflammation–immunity and senescence), and integrative endpoints (epigenetic clocks, multi-omics, functional outcomes, and patient-reported measures). These signals are proposed for integration into a Composite Response Index (CRI) to support prospective evaluation of real-time monitoring and adaptive titration across treatment cycles and clinical scenarios. Evidence is synthesized narratively across HBOT indications, oxygen-biology mechanisms, safety literature, trial-methodology guidance, and biomarker-practicality considerations.

**Results:**

The framework proposes candidate, prospectively testable rules for dose adjustment, phenotype-based stratification, and biologically aligned monitoring schedules. It supports dual-channel stratification by indication and phenotype, enabling hypothesis-driven “right patient, right dose” strategies, and integrates evidence generation across randomized trials, platform studies, and real-world data using causal inference and Bayesian approaches. These operational rules are intended as framework-level proposals and require prospective validation before routine clinical implementation.

**Conclusions:**

This biomarker-driven strategy operationalizes HBOT as a quantifiable intervention, enabling personalized dosing, improved reproducibility, and more consistent clinical responses, with translational potential across neurorehabilitation, cardiometabolic health, wound care, and healthy ageing. The CRI, PTI-based dose mapping, biomarker thresholds, and titration rules should therefore be interpreted as a de novo implementation framework and prospective validation target, not as clinically validated decision limits.

**Supplementary Information:**

The online version contains supplementary material available at https://doi.org/10.1186/s12967-026-08722-w.

## Introduction: from “Disease-centric” to “Biomarker-driven”

### Background and pain points

Hyperbaric oxygen therapy (HBOT) is often guided by a “list of indications plus clinician experience” approach – classically for decompression sickness (DCS), and commonly for wound care, radiation-related soft-tissue injury, ischemia–reperfusion states, and selected neurological conditions. Even when high-quality randomized controlled trials (RCTs), lower-rigor quasi-randomized studies, or specialty guidelines exist, day-to-day care still lacks personalization, biomarker support is limited, and outcomes vary widely. As a result, it remains unclear who benefits, why they benefit, and how to make results repeatable [1–5].

In practice, the optimal combination of pressure – in atmospheres absolute (ATA)—session duration, weekly frequency, and exposure pattern varies by patient. Phenotypes such as high inflammation (particularly the senescence-associated secretory phenotype, SASP), endothelial dysfunction, or a mitochondrial low-energy state may respond differently to the same nominal protocol. Yet “one size fits all” dosing remains common, with little adjustment mid-course. When early biological changes are weak or safety thresholds are being approached, clinicians often lack objective signals to increase, reduce, or pause treatment. This leads to under-dosing for some and avoidable risk for others, compounded by wide protocol variation across trials – highlighting the need for dose standards and response-guided titration [3–10].

Variation also arises from delivery context: chamber type and settings, compression and decompression speeds, air-break plans, oxygen hardware, site workflow, add-on measures (exercise, diet, drugs), and adherence can make the same “pressure × time” biologically non-equivalent. Trial issues – blinding challenges, mixed endpoints, and uneven timing windows – further weaken signal and generalizability. For example, the HBOT for chronic radiation-induced proctopathy (HOT2) trial, a randomized, double-blind, sham-controlled study, found no superiority on its primary endpoints under its chosen protocol. The result is uncertain decisions, fragmented evidence, and limited external generalizability. A better path is to shift from disease-led experience to a biomarker-driven model that uses layered biomarkers, dose-equivalency metrics (e.g., pressure–time integral (PTI), cumulative oxygen exposure), and composite endpoints to actively manage complexity and heterogeneity, improving predictability and reproducibility across centers [3–5, 11–23].

The clinical evidence base also varies substantially by indication. Chronic wound and diabetic-foot evidence includes systematic reviews suggesting potential benefit in selected patients, but estimates differ across ulcer severity, comparator care, follow-up windows, and risk of bias [5–8, 24–27]. Evidence for musculoskeletal pain syndromes also remains limited and heterogeneous, despite signals of potential benefit in recent systematic reviews [28]. In late radiation tissue injury, earlier positive trials contrast with the null HOT2 trial for chronic radiation-induced proctopathy, illustrating how endpoint choice, sham design, tissue phenotype, and timing can change conclusions [2, 29, 30]. In traumatic brain injury and persistent post-concussive symptoms, Cochrane-level reviews and later sham-controlled trials report mixed efficacy signals and highlight blinding difficulty, sham pressure effects, and adverse-event interpretation [31, 32]. These examples support the need for a framework that separates indication-level evidence strength from phenotype-level biological plausibility. In such high-heterogeneity contexts, particularly traumatic brain injury/post-concussive symptoms (TBI/PCS) and longevity-oriented applications, the framework should be interpreted as a research and monitoring structure rather than a validated clinical pathway. Table 1 summarizes the evidence-quality orientation used to position selected indications within the proposed precision HBOT framework.

**Table 1:** Evidence-quality orientation for selected HBOT indications within a precision framework

Evidence domain	Current signal	Main limitations/gaps	Implication for this framework
Established clinical indications and UHMS-style indication lists	Clinical use is supported for selected accepted indications and specialty settings.	Indication lists do not specify individualized dose rules, phenotype selection, or biomarker-guided stopping criteria.	Use indication as the clinical entry gate, but avoid treating it as sufficient for precision dosing.
Chronic wounds / diabetic foot ulcers	Systematic reviews and meta-analyses suggest benefit in selected wounds, particularly when standard care is optimized.	Heterogeneous Wagner grade, vascular status, off-loading, infection control, oxygen protocols, and outcome windows.	Pair wound endpoints with endothelial, redox, inflammatory, and ECM repair markers.
Late radiation tissue injury	Some trial evidence supports benefit in specific radiation injuries, while HOT2 was negative for chronic radiation proctopathy.	Endpoint selection, sham design, tissue phenotype, and timing affect signal detection.	Prespecify tissue-specific endpoints and avoid generalizing across radiation-injury phenotypes.
TBI / post-concussive symptoms	Mechanistic plausibility and some clinical signals coexist with null or mixed controlled evidence.	Blinding, sham pressure, neuropsychological endpoints, and adverse-event interpretation remain challenging.	Use sham-aware designs, objective functional endpoints, and safety monitoring before clinical extrapolation.
Longevity / healthy ageing contexts	Mechanistic and small-study signals exist, but direct clinical outcome evidence is immature.	Commercial enthusiasm may exceed validation; long-term safety and value remain uncertain.	Limit claims to research/monitoring contexts and require transparent conflict-of-interest disclosure and prospective validation.

### From “Disease-centric” to “Biomarker-driven”: a paradigm shift

Precision medicine is not simply “more tests”; it is a controllable input–process–output chain anchored to actionable, reproducible biomarkers. In HBOT, dose inputs – pressure, duration, frequency, and exposure mode – should be explicitly linked to proximal biology (redox status/mitochondrial energetics, nitric oxide (NO)–mediated endothelial and microcirculatory function, inflammation–immunity, extracellular matrix (ECM) remodeling, and neuroplasticity) and then to functional and patient-reported outcomes. When biomarkers inform prescriptions rather than merely documenting them, titration becomes real-time and phenotype-guided, and trials can incorporate adaptive features that respect underlying biology. Platform trials and real-world evidence (RWE) designed with causal inference methods and artificial intelligence (AI) modeling are natural extensions for handling heterogeneity while preserving external validity [11–36].

This represents a shift from disease labels to pathway phenotypes. Individuals can be positioned along axes such as mitochondria–endothelium–inflammation. For example, a high-inflammation/SASP phenotype may benefit from moderate pressure delivered more frequently alongside anti-inflammatory nutrition; an endothelial-dysfunction phenotype should target measurable improvement in flow-mediated dilation (FMD) with concurrent lipid-lowering therapy; and a mitochondrial low-energy phenotype may require moderate pressure with slightly longer sessions to promote biogenesis [15–19, 37–38].

### Why HBOT must be biomarker-driven

Oxygen should be treated as a measurable drug. Nominal prescriptions can be converted into biological inputs using the PTI and cumulative oxygen exposure, while A-layer biomarkers serve as in-course sensors that define each person’s efficacy–safety window [11–19]. Redox panels – F2-isoprostanes, 8-oxo-2′-deoxyguanosine (8-oxo-dG), reduced/oxidized glutathione ratio (GSH/GSSG)—distinguish a beneficial reactive oxygen species (ROS) pulse from overshoot; endothelial readouts (flow-mediated dilation (FMD), reactive hyperemia index (RHI), nitric oxide metabolites (NOx), vascular cell adhesion molecule-1 (VCAM-1), intercellular adhesion molecule-1 (ICAM-1)) indicate whether a microcirculatory threshold has been crossed; mitochondrial signals (oxygen consumption rate (OCR), lactate/pyruvate (L/P), adenosine triphosphate/adenosine diphosphate (ATP/ADP)) reflect activation of energy pathways [11–19]. With these signals, decisions about when to up-titrate, de-escalate, or pause become data-driven: for example, reduce pressure or split exposure if oxidative stress overshoots at 2.4 atmospheres absolute (ATA); modestly increase pressure or extend time if the endothelial response is weak at 1.5 ATA [18, 19].

Beyond HBOT-specific literature, broader experimental work on redox-active and plant-derived compounds further illustrates the biological relevance of oxidative-response modulation and tissue-repair pathways. For example, Pistacia vera L. hull extract has shown wound-healing activity in a rat excision-wound model, while biosynthesized selenium nanoparticles derived from Solanum tuberosum peel extract have demonstrated antioxidant-related biological activity. Phenolic-rich plant extracts, including Myrtus communis fruit extracts, have also been reported to exhibit antioxidant activity. These studies do not constitute direct evidence for HBOT efficacy or biomarker-guided HBOT dosing, but they support the broader biological rationale that redox balance, oxidative-injury control, and wound-repair signaling are important therapeutic and monitoring axes [39–41].

Standardized A/B/C panels and aligned sampling windows reduce heterogeneity and improve reproducibility by enabling dose-equivalence mapping across devices and workflows, using A-layer indicators as a “biometric ruler.” Detailed biomarker stratification and sampling strategies are provided in Supplementary Table S1. The A-layer denotes proximal, dose-titration sensors (redox, mitochondria, endothelium/microcirculation, extracellular matrix (ECM)); the B-layer captures mediators and amplifiers (such as inflammation–immunity and aging-related signatures); and the C-layer comprises integrative downstream endpoints (multi-omics, epigenetic clocks, functional outcomes, and patient-reported outcomes). A Composite Response Index (CRI) replaces single endpoints and pools multi-axis effects into a more stable and portable effect size. Pre-set thresholds, triggers, air-break rules, and early-stop/de-escalation criteria create an auditable chain for regulators and payers. In real-world evidence (RWE), target-trial emulation with propensity methods, doubly robust estimators, and instrumental variables links short-term biomarker gains to mid- and long-term clinical and economic outcomes. Software as a Medical Device (SaMD) and Artificial Intelligence–enabled Medical Device (AI-MD) documentation supports algorithm governance across risk, lifecycle, and change control [20–36].

### Objectives and the question checklist

We propose an operational and prospectively testable HBOT framework organized around five questions: why measure, what to measure, how to use the measurements, how to generate evidence, and how to govern implementation. Biology is structured into three tiers: an A-layer of proximal sensors for candidate dose titration (redox status, mitochondrial function, endothelium/microcirculation, and ECM); a B-layer of mediators (inflammation–immunity and aging-related signatures); and a C-layer of integrative endpoints (epigenetic clocks, proteomics, metabolomics, lipidomics, microbiome, functional outcomes, and patient-reported outcomes). A comprehensive overview of the biomarker layers and their proposed research and implementation roles is provided in Supplementary Table S1.

Operationally, A-layer indicators are proposed to support early assessment, candidate threshold triggers, and a small-step adaptation loop, all of which require prospective validation before clinical use. Dose is expressed as the PTI and cumulative oxygen exposure to derive dose–response curves and stop rules linked to redox, endothelial, and energetic thresholds; these are prespecified in protocols. Efficacy and safety are integrated through a Composite Response Index (CRI) that combines B- and C-layer signals with flow-mediated dilation (FMD), maximal oxygen uptake (VO₂max), cognition, and wound healing metrics. Pulmonary and central nervous system oxygen toxicity and diffusing capacity of the lung for carbon monoxide (DLCO) are monitored, with electroencephalography (EEG) included in selected studies. CRI specifications are preregistered for multicenter validation [33–42].

Evidence generation spans parallel randomized controlled trials (RCTs), platform trials with adaptive randomization, and registry-based real-world evidence (RWE) designed as target-trial emulations. Multi-omics and wearables feed hierarchical Bayes and pathway-aware models to build interpretable predictors, while digital twins with model predictive control (MPC) enable online tuning and decision support [33–36]. Minimum Reportable Biomarker Set (MRBS) and preregistration norms facilitate concordance and secondary analyses [20–23].

### Expected benefits and scope of application

#### Clinical utility

A biomarker-driven HBOT program is proposed to complement experience-based delivery with candidate threshold-guided individualization. In neuro-rehabilitation, cardiometabolic risk, chronic/wound care, and healthy aging, prespecified 8–12-week targets include a 1–2% increase in flow-mediated dilation (FMD) with nitric oxide metabolites (NOx) recovery; a 10–15% rise in oxygen consumption rate (OCR) with a lower lactate/pyruvate (L/P) ratio; and faster wound or extracellular matrix (ECM) healing aligned with matrix metalloproteinase-9 (MMP-9)/tissue inhibitor of metalloproteinases-1 (TIMP-1) and procollagen type III N-terminal propeptide (P3NP). Coupled with a Composite Response Index (CRI) and minimum clinically important difference (MCID), these changes are hypothesized to reduce serious events, shorten treatment courses, and improve maximal oxygen uptake (VO₂max), heart rate variability (HRV), and cognition within routine workflows [11–16, 18–19, 37–38]. These expected benefits remain framework-level hypotheses requiring prospective validation before routine clinical interpretation.

#### Translation

A unified A/B/C panel with harmonized sampling enables cross-center comparability and mechanism-to-dose-to-endpoint mapping. Prespecified CRI and fixed windows boost signal in smaller samples; external validation checks transportability and device/process equivalence. Digital twins and online model MPC transform the data-to-decision-to-evidence loop into deployable pathways and quality key performance indicators (KPIs) [33–36].

#### Boundaries and prerequisites

HBOT benefits and risks vary by equipment, process, adherence, lifestyle, and co-therapies; caution and contraindications include uncontrolled epilepsy, untreated pneumothorax, and severe CO₂ retention. Blinding feasibility, cross-device dose equivalence, and data or model drift can affect effect estimates and generalizability. Scaled use should rely on validated calibration and dose equivalence, rigorous sampling and quality control (including inter-laboratory proficiency testing), preregistered, version-locked CRI analyses, and parallel accumulation of long-term outcomes and economic data so HBOT delivers predictable benefit, managed risk, and value in real-world care [1, 2, 4–6, 9, 10, 20–23].

**Fig. 1:**
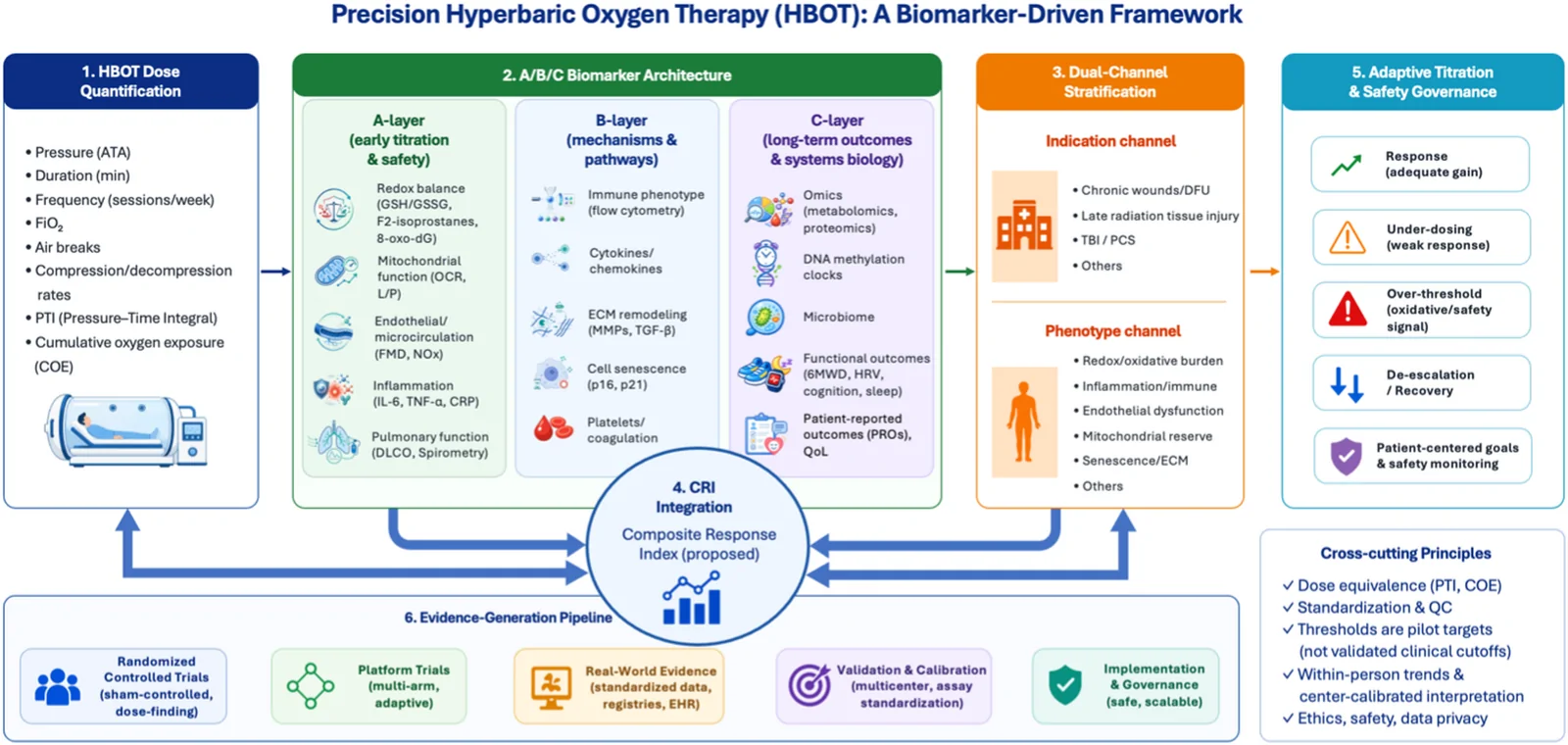
Conceptual framework of precision hyperbaric oxygen therapy (HBOT). The framework integrates dose quantification, A/B/C-layer biomarkers, dual-channel stratification, CRI-based evaluation, and an RCT–platform–real-world evidence pipeline to guide precision HBOT and future validation

The overall conceptual structure of the proposed precision HBOT framework is summarized in Fig. 1.

### Comparison to existing frameworks and innovation

Existing HBOT frameworks primarily organize practice around accepted indications, prescribed pressure and duration, chamber safety, and clinician experience. Prior biomarker papers have described redox, endothelial, inflammatory, neuroplastic, or epigenetic responses, but they generally do not connect these signals to a unified dose-equivalence metric, a tiered decision architecture, or a prespecified titration loop. The incremental contribution of this manuscript is therefore the integration of five elements into one operational pathway: PTI/cumulative oxygen exposure, A/B/C biomarker layering, dual-channel stratification by indication and phenotype, a version-locked CRI concept, and an evidence pipeline linking RCTs, adaptive platform trials, and target-trial-emulated RWE. Table 2 summarizes how the proposed framework differs from existing HBOT approaches.

**Table 2:** How the proposed framework differs from existing HBOT approaches

Component	Conventional or prior approach	Proposed advance in this manuscript
Entry criterion	Disease indication or local clinical protocol.	Indication gate combined with phenotype axes such as inflammation, endothelial dysfunction, mitochondrial low-energy state, and ECM repair profile.
Dose description	Pressure, time, and session count reported separately.	PTI and cumulative oxygen exposure recorded with waveform, air breaks, compression/decompression, and co-interventions.
Biomarker role	Exploratory measurement or post hoc mechanistic interpretation.	Layer A sensors for early titration, Layer B mediators for pathway verification, and Layer C endpoints for durable value.
Endpoint strategy	Single clinical endpoint or unweighted biomarker panel.	Prespecified CRI concept with MCID, missing-data rules, version locking, and external validation plan.
Evidence generation	Single RCTs or narrative accumulation.	Linked RCT, platform-trial, and registry/RWE pathway with causal-inference standards.

## Mechanistic overview: key HBOT axes

### Oxygen sensing, mitochondria, and redox

Hyperbaric oxygen therapy (HBOT) functions less as a static oxygen dose and more as a timed waveform. The intermittent hyperoxia-to-baseline rhythm raises tissue partial pressure of oxygen (pO₂) during the upswing, increasing dissolved oxygen and mitochondrial throughput. This enhances prolyl-hydroxylase (PHD) activity and accelerates hypoxia-inducible factor-1α/2α (HIF-1α/2α) turnover. As pressure and pO₂ return toward baseline, rapid gradient changes are accompanied by low-amplitude, short-lived reactive oxygen species (ROS) pulses that help trigger adaptive transcriptional programs in glycolysis, iron handling, angiogenesis, and antioxidant defense – a suppress→release→re-sensitize sequence rather than blunt HIF suppression [43–46]. This dynamic reframes chronic, low-grade “pseudo-hypoxia” by restoring the responsiveness of oxygen-sensing pathways without extinguishing their capacity to react to genuine hypoxic stressors.

On the energy axis, the steeper oxygen gradient acutely boosts electron transport flux and can engage peroxisome proliferator-activated receptor-γ coactivator-1α (PGC-1α), nuclear respiratory factor-1 (NRF-1), and mitochondrial transcription factor A (TFAM) circuits for mitochondrial biogenesis, while nuclear factor erythroid 2–related factor 2 (Nrf2) activation nudges glutathione and thioredoxin cycling to a higher steady state [15, 47, 48]. These benefits are bounded: if cumulative exposure (pressure × duration × frequency) exceeds tolerance, lipid peroxidation and DNA oxidation increase, coupling efficiency falls, and symptom clusters linked to over-arousal or sleep fragmentation may emerge [47–50]. Early calibration is therefore empirical but measurable. A compact panel – lactate/pyruvate (L/P) ratio, oxygen consumption rate (OCR)/adenosine triphosphate (ATP) coupling, reduced/oxidized glutathione (GSH/GSSG), and F2-isoprostanes/8-oxo-2′-deoxyguanosine (8-oxo-dG)—alongside vascular endothelial growth factor (VEGF), erythropoietin (EPO), and nitric oxide metabolites (NOx), indicates whether oxygen-sensing and energy axes have entered a productive window with oxidative stress controlled [15–56].

### Endothelium and microcirculation

Oxygen delivery and vascular reactivity develop in parallel. Chamber hyperoxia acutely increases plasma-dissolved O₂ and extends diffusion distance in capillary-sparse, edematous, or heterogeneous beds. Intermittency enhances endothelial nitric oxide synthase activity and nitric oxide (NO) bioavailability, reduces vascular cell adhesion molecule-1 (VCAM-1) and intercellular adhesion molecule-1 (ICAM-1), and stabilizes the glycocalyx [45, 53, 57–60]. In ischemia–reperfusion states, higher oxygen tensions can shorten the no-reflow window and reduce microthrombi, smoothing the patchwork of flow that often limits recovery despite adequate upstream perfusion [61, 62]. These improvements are not purely “supply-side”: by reducing leukocyte–platelet adhesion and improving hemorheology, HBOT can lower the inflammatory burden on small vessels and restore perfusion–metabolism matching.

Functional and biochemical readouts are complementary. Flow-mediated dilation (FMD) and reactive hyperemia index (RHI) capture endothelium-dependent dilation at macrovascular and microvascular scales. NOx and adhesion molecules indicate the status of NO–inflammation crosstalk. Imaging modalities such as optical coherence tomography angiography (OCTA), near-infrared spectroscopy (NIRS), and laser-based perfusion methods help distinguish genuine microvascular recruitment from mere oxygen stacking [53–64]. When these signals move together – FMD or RHI up, NOx up, VCAM-1 and ICAM-1 down, and imaging consistent with improved flow heterogeneity – the downstream likelihood of tissue repair increases meaningfully, whether the clinical target is a chronic wound, radiation injury, small-vessel brain disease, or an endothelium-dependent syndrome.

### Inflammation–ECM repair continuum

A recurring challenge in chronic disease is the entanglement of low-grade inflammation with stalled repair. HBOT tends to shift the set point from a high-inflammation/low-resolution background to a low-inflammation/high-resolution steady state through coupled O₂–NO–ROS signaling [38, 65, 66]. The result is not blanket suppression but a repositioning of innate and adaptive circuits: macrophages polarize toward M2, excessive neutrophil extracellular trap (NET) formation is restrained, and leukocyte–endothelium interactions decrease, reducing the traffic that perpetuates microvascular injury [57–67]. On the adaptive side, partial recovery of regulatory T-cell (Treg) function and a more balanced T helper-1/T helper-2/T helper-17 (Th1/Th2/Th17) profile favor orderly healing over scar-dominant trajectories [38, 65, 66]. These shifts explain why symptomatic relief in pain-dominant phenotypes may coincide with objective improvements in endothelial and metabolic measures.

As inflammatory pressure recedes, oxygen becomes structural currency for extracellular matrix (ECM) repair. Oxygen-dependent prolyl and lysyl hydroxylation support collagen stability, while lysyl oxidase–mediated oxidative deamination initiates collagen cross-linking, contributing to ECM maturation and tissue repair. Concurrently, the matrix metalloproteinase-9/tissue inhibitor of metalloproteinases-1 (MMP-9/TIMP-1) balance may shift toward tissue remodeling, while the distribution of hyaluronan may shift toward higher-molecular-weight forms associated with homeostatic signaling and re-epithelialization [68–72]. A practical approach to monitoring this continuum is to combine a small biomarker panel, such as MMP-9/TIMP-1 and procollagen type III N-terminal propeptide (P3NP), with quantitative wound imaging, including area or volume measurements and thermal or perfusion maps. A rising P3NP level that subsequently plateaus as wound edges become more clearly defined, together with a declining MMP-9/TIMP-1 ratio, may indicate progression from proliferation toward remodeling. In contrast, a plateau in wound area accompanied by “hot rims” on thermography may suggest unresolved inflammation or biofilm, indicating that infection control and debridement should be considered before increasing oxygen exposure [63, 64, 73–75]. More broadly, improvements in microcirculation alone may be insufficient to complete tissue repair unless inflammation resolves and matrix organization resumes.

### Neuroplasticity and systems regulation

In the nervous system, the primary contribution of HBOT is to improve local perfusion–metabolism matching in hypoperfused or metabolically constrained regions, thereby creating the substrate for network rewiring. Time-sequenced increases in brain-derived neurotrophic factor (BDNF), nerve growth factor (NGF), glial cell line-derived neurotrophic factor (GDNF), and synaptic plasticity genes are frequently described alongside improved aerobic metabolism, which together facilitate dendritic spine recovery, receptor clustering, and compensation within injured networks [76–79]. These central effects interact with systemic regulators: reduced inflammatory drive, improved vagal tone, and gut–immune–brain crosstalk lower central “noise,” often manifested as steadier mood, reduced attention variance, and more restorative sleep [80–83]. Clinically, this oxygen → plasticity → rhythm chain is consistent with observations in post-traumatic brain injury, post-stroke cognitive impairment, brain fog–like states, migraine biology, and patterns of autonomic dysregulation.

Calibration can remain pragmatic. Brief task batteries for attention and working memory, sleep architecture from wearables, and heart rate variability (HRV) metrics provide proximal signals of benefit or over-arousal; composite cognitive scores and psychometrics quantify durability; and quantitative electroencephalography (qEEG) or perfusion imaging can be reserved for research contexts or difficult cases [79–83]. When reaction time gains are accompanied by healthier REM/N3 balance and improved HRV (e.g., higher root mean square of successive differences (RMSSD) with a lower low-frequency/high-frequency (LF/HF) ratio), the intervention is likely below the arousal threshold that destabilizes sleep. If not, shortening sessions, adding air-break segmentation, or shifting to earlier daytime slots usually restores equilibrium.

### Coupling, temporal hierarchy, and composite readouts

These axes are interconnected and unfold on different time scales. Proximal signals in redox, nitric oxide biology, and mitochondrial flux often shift within 1–2 weeks; inflammatory mediators, adhesion molecules, immune subset patterns, and extracellular matrix activity consolidate over 2–6 weeks; integrative endpoints – maximal oxygen uptake (VO₂max), heart rate variability, sleep structure, cognition composites, wound healing rates, and even epigenetic clock deceleration – emerge over 8–12 weeks and beyond [38, 65, 73–78, 80–83]. Because gains on one axis erode when others do not co-move, coordinated assessment windows are important: tune the waveform against proximal sensors early, verify pathway engagement mid-course, and confirm functional value later. This temporal layering also cautions against overinterpreting any single early laboratory shift as definitive success or failure.

For the same reason, no single marker can represent net benefit. A composite response that combines proximal and pathway-level shifts (A + B ± C) is typically more robust and more portable across centers. Weighting can be anchored in biological plausibility, correlation with functional outcomes, and reproducibility, with mediation analyses separating direct effects from pathway-mediated effects so that “oxygen piling without utilization” is not mistaken for “no effect” [35–85]. Framed this way, the dosing chapter that follows can treat oxygen as a measurable medicine – titrated against proximal sensors, verified by pathway closure, and confirmed on integrative outcomes – without overloading the mechanistic narrative with operational details. The result is a tractable, reviewable path from waveform to biology to function that aligns clinical pragmatism with translational rigor.

## Precision HBOT: mechanisms, outcomes, and operations

### A-layer: proximal sensors for real-time dose titration

The A-layer functions as an online sensor to maintain dosing within protocol-defined candidate efficacy and safety boundaries using rapid, reproducible assays [86–88]. Representative biomarkers, sampling schedules, and clinical applications are detailed in Supplementary Table S1. The numerical examples in this section should not be interpreted as established consensus thresholds or clinically validated decision limits; rather, they are candidate, protocol-defined triggers for prospective evaluation. Safety is monitored by oxidative stress markers (F2-isoprostanes, 8-oxo-dG, GSH/GSSG) under standardized sampling; in prospective protocols, sustained increases of approximately 20–30% in damage indices, particularly when accompanied by decreasing GSH/GSSG, may indicate excessive oxidative burden and could trigger evaluation of shorter sessions, segmented exposure with air breaks, or lower pressure [87–91]. Efficacy is assessed on energy and endothelial axes: platelet/peripheral blood mononuclear cell (PBMC) OCR and lactate: pyruvate (L/P) reflect oxidative phosphorylation (OXPHOS) mobilization as candidate response signals; for example, OCR increases of approximately 10–15% with decreasing L/P may suggest favorable mitochondrial engagement, while NOx, VEGF, and VCAM-1/ICAM-1 combined with FMD/RHI indicate candidate endothelial response patterns, such as FMD increases by approximately 1–2% points or RHI approximately by 0.1–0.2) [15–52, 54–56, 92–94]. However, these numerical values should be regarded as plausible pilot thresholds derived from measurement conventions and mechanistic expectations rather than validated HBOT dosing thresholds, and they require indication-specific prospective validation before being used for clinical titration. If FMD/RHI do not improve and adhesion molecules do not decline – a candidate pattern consistent with “oxygen exposure without pathway engagement” **—** protocol-defined studies may evaluate increased intermittency or reduced intensity before testing changes in frequency or pressure; co-treatments, including exercise, statins, and PCSK9 inhibitors, should be documented [95, 96]. A minimal viable panel for prospective monitoring rather than routine clinical titration includes: (i) F2-isoprostanes and 8-oxo-dG; (ii) NOx and FMD/RHI; (iii) L/P and sentinel OCR, sampled at baseline, week 2, weeks 4–6, and weeks 8–12; early weekly checks (weeks 1–3) are useful in high-risk or strongly responding cases [54, 86, 97]. Accordingly, the A-layer is intended to provide a biologically informed monitoring structure for future studies, not a set of validated clinical dose-adjustment rules.

Because several A-layer assays show inter-laboratory variability, dose decisions should not rely on single uncalibrated values. Each center should establish baseline calibration using duplicate baseline samples, standardized fasting morning collection, fixed post-session sampling windows, common reference materials where available, pooled internal controls, and external proficiency testing. For multicenter studies, marker values should be normalized as within-person percentage change and center-calibrated Z-scores before entering titration rules or CRI calculations; absolute cutoffs should be avoided unless assay equivalence has been demonstrated.

### B-layer: pathway readouts and combination guidance

The B-layer translates proximal changes into pathway evidence, guiding mid-course strategy and adjuncts (exercise, nutrition, photobiomodulation, drugs) at week 4–6 and week 8–12 checkpoints [97, 98]. On the inflammation–SASP axis, declines in IL-6/TNF-α (≥ 20–30%) or CRP normalization indicate successful de-inflammation; if there is no improvement, first back-trace A-layer over-threshold signals (oxidative peaks, sleep/HRV deterioration, no FMD gain), then add ω-3/Mediterranean nutrition and sleep hygiene, and consider more intermittency or lower intensity to avoid peak stacking [88, 98–100]. Immune phenotyping (Treg/Teff balance, Th1/Th2/Th17, NK activity, NLR) indicates whether repair or regeneration is favored; persistent Th17 skew or MMP-9/TIMP-1 dysregulation suggests a fibrosis-prone course and supports a conservative dose profile with extended recovery [38, 65, 70–72]. Research-grade senescence readouts (p16^INK4a, exosomal senescence markers) can be included in longer courses or healthy-aging programs but should not drive clinical titration alone [101].

### C-layer and Composite Response Index (CRI): from biology to durable benefit

The C-layer captures durable, system-level value for RCTs and RWE: DNA methylation clocks, including GrimAge and PhenoAge as reference measures, for assessing changes in biological-age estimates and age acceleration; targeted proteomic, metabolomic, and lipidomic analyses for cardiometabolic risk trajectories; microbiome features, including short-chain fatty acid (SCFA) capacity, barrier function, and lipopolysaccharide (LPS)/trimethylamine N-oxide (TMAO) pathways, for gut–immune–metabolic links; and functional outcomes, including VO₂max, HRV, sleep architecture, 6-minute walk distance (6MWD), grip strength, and standardized cognitive composites, aligned with patient-reported benefit [21, 97–104]. These inform a prespecified CRI that integrates the A-layer (immediate biologic effect), B-layer (pathway activation or closure), and C-layer (functional value) into a single continuous metric for prospective evaluation of early go/no-go decisions, dose–response modeling, and cross-center comparison [104]. Directions should be standardized, with improvement coded as positive, using Z-scores and predefined MCID and missing-data plans. Initial candidate weights may be provisionally set at A: 40–50%, B: 25–35%, and C: 20–30%, with external validation and version locking required [20, 104–106]. In prospectively defined protocols, weeks 2–4 may serve as an early assessment window. If CRI ≥ MCID, consolidation or optimization of adjuncts may be evaluated; otherwise, candidate adjustments may be tested sequentially, beginning with duration and intermittency, followed by pressure or frequency. CRI may then be modeled against PTI and cumulative O₂ exposure to explore potential “over-threshold” states (a flat CRI accompanied by increasing oxidative-damage markers) and “under-dosed” states (a slowly increasing CRI accompanied by subthreshold endothelial responses) [107, 108].

Validation status. The CRI and example thresholds in this manuscript are proposed targets for protocol development and prospective validation; they have not been retrospectively validated against a harmonized multicenter HBOT dataset and should not be interpreted as validated clinical decision limits. The numerical examples, such as a 20–30% increase in oxidative-damage indices, a 1–2%-point FMD gain, a 0.1–0.2 RHI increase, a 10–15% OCR increase, or a 20–30% decline in inflammatory markers, should be treated as plausible pilot thresholds derived from measurement conventions, biological plausibility, and mechanistic expectations rather than as peer-reviewed consensus thresholds for HBOT dosing. Future studies should estimate indication-specific MCIDs, evaluate calibration and discrimination, assess within-person and between-center variability, and report whether CRI changes predict patient-centered outcomes beyond standard clinical covariates. Until such validation is completed, CRI should be used as a research, registry, or protocol-monitoring construct rather than as a stand-alone tool for clinical dose adjustment.

### Operations: sampling/QC, tiered monitoring, and follow-up milestones

Comparability depends on pre-analytical factors (fasting morning blood, first-void urine, caffeine and exercise embargoes) and controlled conditions for FMD/RHI (thermoneutral room, rest period), with deviations recorded as metadata [54, 56, 82]. Limit batch effects through randomized plate positions, pooled QC and external references, Levey–Jennings charts with Westgard rules, cross-site comparisons, and mixed-effects calibration; lock equivalence and conversion formulas in the methods appendix [20, 23, 109]. Favor clinically robust platforms (immunoassays, targeted LC-MS/MS) for decision panels; reserve multi-omics for research or master protocols with prespecified identification and false discovery rate (FDR) thresholds and statistical analysis plan (SAP)-level validation to prevent misuse as clinical decision variables [20]. Build a unified data and metadata model and auditable pipelines (variable dictionary, units, missingness semantics, device, lot, operator, timestamps, pre-analytics), enable tiered access and audit logging, and consider federated learning for multi-center modeling; handle missingness and outliers with multiple imputation and robust summaries plus sensitivity analyses [21–23, 110].

Monitoring can be tiered under one QC schema: Basic (A-layer minimal panel every 2–3 weeks plus wearables and brief function tests) [80, 97]; Standard (complete A-layer, two B-layer checkpoints, and C-layer at 12 weeks and 6 months with predefined primary and key secondary endpoints) [20, 105, 109]; Enhanced (add ECM and aging markers and selected omics; increase A-layer to weekly in weeks 1–4 to support adaptive randomization) [97, 105].

For routine non-research care, the minimum viable implementation should be deliberately lighter than the platform-trial schedule. The lowest-cost implementation tier may rely on structured dose logs, PTI/cumulative oxygen exposure calculation, symptom and adverse-event checklists, basic pulmonary/ENT screening, wearable-derived HRV and sleep summaries, 6-minute walk distance, grip strength, wound photography, patient-reported outcomes, and selected routine laboratory markers. A pragmatic starting panel may include symptom and adverse-event checklist, basic pulmonary/ENT screening, FMD or RHI where available, NOx or an endothelial surrogate, urinary F2-isoprostanes or 8-oxo-dG, lactate/pyruvate or a simple metabolic surrogate, HRV/sleep monitoring, and one indication-specific functional endpoint. Enhanced OCR, detailed redox panels, flow cytometry, omics, DNA methylation clocks, and microbiome testing should be reserved for trials, specialty programs, or high-risk cases until cost-effectiveness, reimbursement, turnaround time, and personnel training requirements are clarified.

Recommended milestones: Baseline → Week 2 (optional in high-risk or trial cohorts) → Week 4–6 → Week 8–12 → Month 3–6, with A-layer re-tests at least twice in the first 4–6 weeks (weekly in weeks 1–3 for strong responders or high-risk), B-layer at weeks 4–6 and 8–12 (plus an early week 2 or 3 checkpoint for high inflammation or immune imbalance), and C-layer at weeks 8–12 and months 3–6. If any node flags safety (oxidative surge, DLCO drop, CNS prodrome) or efficacy stall (CRI < MCID), trigger unscheduled re-testing and titration review to return to the individualized efficacy–safety corridor [21, 108].

### Clinical readiness, feasibility, turnaround time, and health-economic considerations

The three biomarker layers differ substantially in clinical readiness. Routinely accessible elements include structured dose logs, PTI and cumulative O₂ exposure calculations, symptom and adverse-event checklists, pulmonary and ear–nose–throat (ENT) screening, wound photography, selected routine laboratory markers, wearable-derived HRV and sleep summaries, 6-minute walk distance (6MWD), grip strength, and patient-reported outcomes. Some A-layer measures, such as FMD/RHI, urinary F2-isoprostanes or 8-oxo-dG, NOx, and lactate/pyruvate (L/P), may be feasible in specialized centers but require standardized sampling windows, operator training, assay calibration, and local turnaround-time validation before being used for protocol decisions. In contrast, OCR, detailed redox panels, flow cytometry, senescence markers, DNA methylation clocks, targeted proteomics, metabolomics, lipidomics, and microbiome profiling should be considered advanced research or registry-level tools rather than routine clinical titration instruments until prospective clinical utility is demonstrated [54, 80, 109].

The proposed 24–72-hour “rapid A-layer” screen should therefore be interpreted conservatively. In routine practice, it is most realistic when based on immediately available exposure data, safety signals, symptom trajectories, wearable summaries, brief functional tests, wound imaging, and selected targeted assays with validated local turnaround. Multi-omics, DNA methylation clocks, and most targeted proteomic or microbiome assays usually require batching, specialized workflows, and longer reporting times, making them more appropriate for baseline stratification, trial endpoints, or registry follow-up rather than short-cycle dose titration.

A tiered implementation strategy may be more feasible than universal deployment of the full biomarker framework: a low-cost minimum dataset for routine documentation, a standard registry dataset for cross-center comparability, and an enhanced research dataset for mechanistic and platform trials. Health-economic evaluation should capture assay costs, staff time, equipment needs, quality-control requirements, reimbursement feasibility, patient burden, avoided adverse events, treatment duration, wound-healing time, hospitalization, return to work, quality of life, and budget impact [20, 104]. Until such evidence is available, biomarker-guided HBOT should be presented as a prospectively testable implementation framework rather than a proven cost-effective clinical strategy.

## Dosing the prescription: making O₂ a measurable medicine

HBOT dosing should transform oxygen from an environmental condition into a quantified input, a monitorable process, and a predictable output. Core variables are pressure (ATA), session duration (minutes), weekly frequency (sessions per week), total course length (weeks), and exposure architecture (continuous vs. intermittent, with or without air breaks), with time-stamped records of concomitant interventions (exercise, nutrition, drugs, photobiomodulation) for causal interpretation [24, 111]. To enable comparison across devices and centers, express dose with two harmonized metrics: (i) pressure–time integral (PTI = ATA × minutes) and (ii) cumulative oxygen exposure (PTI × %O₂, summed across sessions). When FiO₂ is approximately 1.0, these metrics converge; with air breaks or FiO₂ less than 1.0, cumulative exposure better reflects the biological stimulus [24, 111]. Detailed dosing parameters, safety monitoring indicators, and titration strategies are summarized in Supplementary Table S2.

For example, a 90-minute session at 2.0 ATA has a nominal PTI of 180 ATA-min, whereas a 60-minute session at 2.4 ATA has a nominal PTI of 144 ATA-min; however, the latter may generate higher peak oxidative and CNS-arousal risk because waveform amplitude and air-break structure differ. Therefore, PTI should be interpreted together with FiO₂, air-break timing, compression/decompression rate, and proximity to A-layer safety thresholds rather than as a stand-alone equivalence formula.

Dose–response should be linked to proximal (“A-layer”) candidate thresholds and kinetics, with all thresholds treated as protocol-defined targets for prospective validation. If a 2.4-ATA continuous pattern produces rising F2-isoprostanes with falling GSH/GSSG, this pattern may indicate a transition from beneficial ROS signaling toward oxidative damage; prospectively defined protocols may therefore test pressure reduction (e.g., to approximately 2.0 ATA) or segmented exposure (e.g., 5–10-minute air breaks or two shorter exposure periods) [90, 91]. If no improvement in FMD/RHI or NOx is observed at 1.5 ATA, protocol-defined studies may evaluate whether extending exposure duration or increasing pressure to 1.7–2.0 ATA produces an endothelial response without exceeding safety limits [54, 93]. Phenotypes may inform candidate starting strategies: low mitochondrial energy, characterized by low OCR and high L/P, may be evaluated using moderate pressure (1.5–2.0 ATA) with longer sessions, whereas acute microcirculatory or antimicrobial indications may warrant investigation of short-term higher pressure (2.4–2.5 ATA) with strict air-break procedures and closer safety monitoring [95, 96, 101]. At an equivalent PTI, waveform may influence biological responses: continuous high-intensity exposure may produce higher ROS peaks and CNS arousal, whereas intermittent or segmented exposure may reduce toxicity risk and modulate HIF, endothelial, and immune-resolution pathways. For individuals with poor sleep or high sympathetic tone, prospective protocols may evaluate earlier scheduling and intermittent exposure. For high-inflammation or chronic-wound phenotypes, moderate-pressure, higher-frequency regimens (4–5 sessions per week) should be prospectively compared with sporadic high-pressure regimens, while the potential contributions of diet and protein intake to ECM remodeling should be documented as relevant co-interventions [80, 99].

Protocol-defined safety boundaries are integrated into the proposed dosing framework. Monitor pulmonary oxygen toxicity using spirometry and DLCO, along with oxidative damage panels (F2-isoprostanes, 8-oxo-dG); persistent cough, chest tightness, or a decline in DLCO warrants reducing pressure or duration and adding air breaks and recovery days [89, 91]. CNS oxygen toxicity is primarily a clinical diagnosis (visual or sensory phenomena, nausea, agitation, perioral twitching, prodromal myoclonus); at any prodromal sign, decompress or stop, breathe air, then down-titrate PTI and shorten subsequent continuous exposures. Consider drug interactions (CNS stimulants, certain antibiotics) and CO₂ retention or ventilatory deficits that increase CNS excitability or disrupt acid–base balance [24, 90].

Titration uses the A-layer as an “online radar.” Candidate titration rules and protocol-defined monitoring thresholds for prospective validation are further detailed in Supplementary Table S2. Within the proposed framework, dosing should aim to remain between candidate efficacy thresholds (FMD/RHI increase, OCR increase, L/P decrease) and safety thresholds (F2-isoprostanes/8-oxo-dG increase, GSH/GSSG decrease, symptom flags) [54, 86, 92]. A practical sequence is: Weeks 0–2/3—micro-adjust pressure and duration in small steps (± 0.2–0.3 ATA or ± 10–15 min) and add or extend air breaks first; Weeks 4–6—reconcile A- and B-layer evidence to decide whether to maintain, consolidate, switch arms, or down-titrate [97, 112]. Frequencies should match biology: endothelium and microcirculation typically require 4–5 sessions per week for 8–12 weeks; mitochondrial endurance can improve with 3–4 sessions per week; sleep and mood endpoints emphasize avoiding late-evening high intensity and preserving recovery days (e.g., 3–4 sessions per week, alternate days) [54, 97].

Operationalization ensures cross-center equivalence. Each prescription should record PTI and cumulative O₂ exposure, air-break timing and duration, compression–decompression rates, and in-chamber activity limits to enable dose-equivalence modeling and audit [80, 111]. For combinations, predefine relative timing: schedule aerobic exercise approximately 4–6 h after HBOT to avoid overlapping ROS peaks and to exploit a plasticity window; schedule resistance training and red/NIR on alternate days; log statins/PCSK9i, GLP-1RA/SGLT2i, and antiplatelet/anticoagulants to interpret B-layer signals [95, 99]. Pair every plan with early-stop rules (any symptom or A-layer over-threshold → stop or downgrade), fixed review points (Week 2, Weeks 4–6, Weeks 8–12), and a 3–6-month follow-up for C-layer endpoints so the measure–treat–evaluate loop remains executable, traceable, and reviewable in practice [21, 105].

## Population stratification: dual channels of indication × phenotype

The proposed precision HBOT framework is organized around two parallel entry channels. The indication-based (disease-type) channel anchors enrollment to recognized or study-defined clinical conditions and associated safety boundaries, whereas the phenotype-based (geroscience-type) channel is intended to explore whether molecular and systems-level features can help explain response heterogeneity and identify potentially modifiable pathways [25, 30, 111]. Disease-type cohorts may include non-healing or diabetic foot wounds, radiation-induced soft-tissue injury, post-ischemia–reperfusion states, post-TBI syndromes, and cardiometabolic high-risk populations. However, the strength and consistency of evidence vary substantially across these conditions, particularly for post-TBI syndromes and cardiometabolic applications [25, 30, 32, 101]. The phenotype channel cuts across diagnostic categories and may include high inflammatory or SASP activity, mitochondrial low-energy status (high lactate or low VO₂max), endothelial dysfunction, ECM fibrosis propensity, accelerated DNA methylation (DNAm) age, and sleep or psychosocial impairment. These features are hypothesized to map onto candidate O₂–NO–ROS–energy–ECM response axes and require prospective validation before being used for treatment selection or dose adjustment [88, 98].

Operationally, a “rapid A-layer” screen establishes mobilizability and preliminary thresholds within 24–72 h: urinary F2-isoprostanes plus 8-oxo-dG for oxidative-stress gating, plasma NOx with FMD/RHI for endothelial thresholds, and lactate/pyruvate (L/P) with an index OCR for the energy axis [54, 86, 87]. When the rapid panel indicates threshold engagement or high mobilizability, the formal course begins, with pathway verification at weeks 2–3 and 4–6 via B-layer readouts (inflammation–immunity panels, adhesion molecules, flow-cytometry subsets). Adjuncts – exercise, nutrition, red/near-infrared photobiomodulation, and indicated medications – are scheduled to amplify the targeted axes [95, 97, 99]. For mid- to long-term value or study inclusion, C-layer endpoints (DNAm age, targeted proteome/metabolome/lipidome, microbiome, and functional/psychological outcomes such as VO₂max, HRV, sleep architecture, cognition, and mood scales) are assessed at weeks 8–12 and months 3–6 as primary or secondary endpoints and to stabilize the Composite Response Index (CRI) in trials and RWE [20, 21, 104].

Within disease-type strata, phenotype layering narrows the dosing window and improves predictability. In chronic wounds/diabetic foot, elevated MMP-9/TIMP-1 with active P3NP and persistently high IL-6 often favor moderate pressure with higher weekly frequency in an intermittent structure, plus ECM support and anti-inflammatory nutrition. If endothelial dysfunction coexists (low FMD/NOx), cautious time extension or small pressure uptitration within safety bounds, paired with statins/PCSK9i and structured walking, is reasonable [95, 97–99]. In radiation soft-tissue injury, inflammation-dominant patterns favor segmented protocols to avoid ROS peak stacking, whereas microcirculatory-dominant patterns may justify earlier pressure elevation under close monitoring [97, 99, 101]. For post-TBI or post-stroke cognitive impairment, track sleep architecture, HRV, and mood in parallel; avoid late-evening high-intensity exposure and monitor for CNS excitability prodromes [78, 82, 90].

Three pragmatic (non-exclusive) responder phenotypes are common: (1) high-inflammation (IL-6, TNF-α, CRP high; Th17 skew), emphasizing moderate pressure with higher frequency plus anti-inflammatory adjuncts; (2) endothelial-deficit (low FMD, low RHI, low NOx, high VCAM-1, high ICAM-1), prioritizing a detectable FMD threshold with lipid-lowering and anti-adhesion strategies; and (3) mitochondrial low-energy (low OCR, high L/P, fatigue, low endurance), favoring moderate pressure with longer duration and rhythmic aerobic or resistance training [54, 95–97, 99]. For accelerated DNAm age or psychosocial impairment, HBOT functions mainly as a “baseline pathway tuner” and requires synergy with sleep–mood interventions and social support to achieve durable C-layer gains [20, 21, 104].

A three-step cadence enhances executability: Recruit (indication gate; A-layer rapid panel to set mobilizability and initial safety bounds), Follow-up (weeks 2–6 A/B-layer cycles with small-step titration; once CRI ≥ MCID, consolidate and optimize adjuncts), and Maintain (weeks 8–12 decision on maintenance or consolidation; at months 3–6, evaluate C-layer durability and relapse risk) [21, 97, 104]. Non-responders or intolerant participants are de-escalated, switched, or stopped per A-layer thresholds and adverse event trajectories, with all paths feeding RWE to continuously calibrate rules [21, 105]. Inclusion prioritizes clear indications or high-risk phenotypes, ability to complete key follow-ups, and recordable co-interventions; exclusions and cautions include active epilepsy or uncontrolled CNS excitability, severe CO₂ retention or uncorrected pneumothorax, major recent medication changes, and poor adherence [90, 97]. Through dual-channel entry, rapid A-layer screening, and rhythmic B/C-layer verification, precision HBOT operationalizes the “right person × right dose × right combination” loop, improving consistency, limiting non-beneficial exposure, and providing a replicable stratification template for platform trials and policy decisions [21, 97, 105].

## Study design and methodology

### Randomized controlled trials (parallel-group; superiority/dose-finding)

To prospectively evaluate precision HBOT beyond single-arm evidence, we propose a three-arm, parallel-group RCT comprising conventional HBOT at 2.0–2.5 ATA, mild hyperbaric oxygen therapy (mHBOT; 1.3–1.5 ATA), and a sham-control condition designed to reproduce the chamber experience while minimizing therapeutic hyperoxic exposure [97, 102, 105, 113]. Blinding credibility would be central to the design. Where technically feasible, study arms should use comparable chamber entry and exit procedures, equipment, session duration, communication, and sensory cues, while avoiding physiologically meaningful oxygen exposure in the sham arm. The protocol should prespecify assessment of blinding success using treatment-allocation guess questionnaires completed by participants and relevant study personnel [105, 113]. Eligibility should be anchored to clearly defined indications or prospectively specified high-risk phenotypes and the ability to complete assessments at baseline, weeks 4–6, weeks 8–12, and months 3–6. Exclusion criteria should include uncontrolled epilepsy, untreated pneumothorax, severe carbon dioxide retention, and other protocol-defined conditions associated with unacceptable HBOT risk [111].

The primary outcome may be either a prespecified Composite Response Index (CRI) integrating weighted A-, B-, and C-layer components or a single clinically or functionally meaningful endpoint with strong mechanistic anchoring, such as FMD, VO₂max, a standardized cognitive composite, or wound-healing velocity [54, 80, 104]. Secondary outcomes may include changes in epigenetic-age measures, targeted proteomic and metabolomic scores, HRV and sleep metrics, and patient-reported outcomes, with an a priori endpoint hierarchy and multiplicity-control strategy specified in the statistical analysis plan (SAP) [20, 104, 109]. Sample size should be determined by the prespecified MCID and variance of the primary outcome. For planning a standardized CRI, an effect size of approximately 0.35–0.50 SD may be considered as a provisional range, but this assumption should be refined using pilot data. Stratified randomization by indication and prospectively defined phenotype may improve balance, with appropriate allowance for attrition, clustering, and center effects [97, 102, 112]. The primary analysis should follow the intention-to-treat (ITT) principle, with a per-protocol (PP) analysis conducted as a sensitivity analysis. Continuous outcomes may be analyzed using linear or mixed-effects models, with center- and participant-level random effects where appropriate; generalized or robust models should be prespecified for non-normal or skewed outcomes [105, 109, 114]. Missing data should be addressed using multiple imputation, accompanied by complete-case and best- and worst-case sensitivity analyses [109]. If an interim analysis is planned, its timing and stopping boundaries should be prespecified. An analysis at approximately 50–60% of the planned information, using an O’Brien–Fleming or Pocock-type alpha-spending approach, may improve trial efficiency, while a prospectively defined futility criterion, such as conditional power below 20%, may limit non-beneficial exposure [112]. Safety oversight should include an independent Data and Safety Monitoring Board (DSMB), protocol-defined pulmonary and central nervous system oxygen-toxicity assessments, candidate oxidative-damage guardrails such as F2-isoprostanes and 8-oxo-dG, and emergency-unblinding procedures [111]. Before enrollment of the first participant, investigators should finalize a device and dose-equivalence manual, operator-training requirements, and standardized capture of PTI, cumulative O₂ exposure, air-break structure, compression and decompression rates, and in-chamber activity as core metadata. Prospective trial registration, SAP finalization, and version control of standard operating procedures (SOPs), algorithms, and analysis scripts should follow CONSORT and ICMJE expectations [105].

### Platform trials (Master protocol; adaptive randomization/Bayesian backbone)

Complex combinations of pressure, duration, frequency, intermittency, and co-interventions are best evaluated within a single Master Protocol that standardizes eligibility, follow-up, quality control, and data standards across evolving arms [97, 102, 115]. A rapid A-layer panel (oxidative stress, endothelial response, and energy-axis indicators) serves as an early biologic sensor, while a fixed set of functional endpoints (e.g., FMD, VO₂max, wound-healing velocity, cognitive composites) provides mid- and late-phase confirmation. Sampling SOPs, QC thresholds, variable dictionaries, and device calibration are locked to ensure cumulative and auditable dose–effect–endpoint comparisons [23, 54, 105].

Response-adaptive randomization (RAR) operates through early go/no-go gates: at weeks 2–4, arms that cross prespecified biologic thresholds (e.g., CRI_A gain or FMD rise) receive higher subsequent allocation; arms that repeatedly miss thresholds or trigger safety limits are down-weighted or stopped [103, 112]. Allocation floors and ceilings prevent extremes and protect small or high-risk subgroups, while phenotype-balanced minimization maintains comparability across high-inflammation, endothelial-deficit, and low-energy states [97–103]. Statistical inference uses Bayesian hierarchical models with center and phenotype random effects for principled information borrowing; commensurate priors are used where mechanisms align, with borrowing reduced when they do not [103]. Design-stage simulations (accrual patterns, drift, missingness, measurement error) calibrate type I error, power, stop probabilities, and time to decision, and stress-test adaptive rules [97, 103].

Regulatory-facing elements are prespecified: the integrated diagnostic (composite biomarkers) plus prescription strategy evaluand; adaptive triggers and thresholds; and clear entry, graduation, and stop criteria (e.g., posterior probability thresholds sustained across analyses and free of safety flags) [22, 110]. Shared controls and fixed data or algorithm versions (including a locked CRI formula and missingness strategy) maximize efficiency and reproducibility; an independent statistical center and auditable scripts support full replayability [105]. A standing DSMB monitors tiered safety thresholds (pulmonary/CNS oxygen toxicity, DLCO decline, oxidative-damage surges), and multiplicity is addressed through Bayesian layered posterior thresholds, supplemented when required by frequentist procedures for family-wise control [105, 111, 112, 114]. Patient-centered features, such as controlled cross-over after interim superiority and bounded preference-concordant allocation within RAR limits, improve acceptability without compromising inference [97, 109].

### Real-world evidence (prospective registries; target-trial emulation)

To maximize generalizability and long-term interpretability, multicenter prospective registries should capture a simplified A-layer panel and a small set of functional endpoints on fixed schedules (8–12 weeks; 3–6 months), along with complete dose logging (PTI, cumulative exposure, air breaks) and co-intervention timing (exercise, nutrition, red/NIR, lipid-lowering and metabolic drugs) [21, 35, 109]. Each record includes device and firmware identifiers, compression and decompression rates, in-chamber activity limits, and precise sampling-to-exposure timestamps to support downstream exposure–response modeling and center calibration [23, 110].

Causal inference follows modern observational standards: propensity-score methods for baseline comparability; interrupted time-series or instrumental variables where policies or thresholds create natural experiments; and, for time-varying treatment and confounding, marginal structural models or G-methods [35–59]. Negative-control outcomes and exposures, as well as sensitivity analyses (e.g., E-values, weight trimming, alternative windows), address residual confounding. Hierarchical Bayesian models allow partial pooling across heterogeneous centers while maintaining robustness [103]. To reduce researcher degrees of freedom, analysis plans are pre-registered under a target-trial framework that fixes eligibility, index date, dose and exposure definitions, comparators, endpoints, and windows before analysis [21, 35–85]. Digital follow-up is standardized (device model and firmware, sampling rate, scoring algorithms), with dashboard-based data quality monitoring for missingness, noise, and adherence. When safety limits or plateaus are detected in A-layer or functional endpoints, the system can provide micro-titration suggestions (e.g., − 0.2 ATA, add air breaks, extend recovery days) within routine care pathways [107, 108]. Governance includes operator credentialing, inter-center proficiency testing, de-identification and audit trails, tiered access, and version-locked algorithms and composites within containerized, reproducible environments to ensure every step is traceable from raw data to conclusions [22, 23, 110].

Summary. RCTs provide causal anchors; platform trials accelerate evaluation of multi-parameter regimens under a single standard; and RWE supplies external validity, durability, and health economics relevance. Linked by a unified biomarker system, dose logging, and shared data standards, the three streams form a coherent evidence pipeline in which the A-layer functions as the early gate and titration sensor, B/C-layers provide confirmatory endpoints and the CRI backbone, and all outputs are auditable and regulator-ready [21, 22, 35].

## Statistics and AI: making the data clearly show who benefits

### Data foundations and feature construction

Statistics and artificial intelligence (AI) serve as an analytical bridge linking A/B/C-layer biomarkers with dose records and functional endpoints. A unified variable dictionary, harmonized units, complete sampling and exposure timestamps, and device, operator, and assay metadata should support auditability and reproducibility. Key pre-analytical conditions, including fasting status, recent exercise, caffeine intake, medication use and timing, should be recorded alongside chamber parameters, such as compression and decompression rates and air-break structure, and relevant co-interventions, including statins, glucagon-like peptide-1 receptor agonists (GLP-1RAs), sodium-glucose cotransporter-2 inhibitors (SGLT2 inhibitors), exercise, and red or near-infrared (NIR) light exposure. Missing data and outliers should be handled according to a prespecified protocol. Multiple imputation may be used for missing primary-outcome or covariate data when its assumptions are appropriate, while robust estimators may be applied to skewed or heavy-tailed distributions. Synchronized raw, cleaned, and analysis-ready datasets should be retained to preserve end-to-end traceability [23, 105, 109].

### Prediction and causal inference – two tracks, one aim

For prediction of who may benefit, analyses should begin with regularized linear models, including L1 and L2 regularization and elastic net, before evaluating tree-based ensembles for nonlinear relationships. Hierarchical Bayesian models may account for center- and phenotype-level heterogeneity while providing credible intervals. For multimodal data, graph-based or pathway-informed models may organize biomarkers along mitochondrial, endothelial, and inflammatory axes and generate a candidate, interpretable “HBOT response vector” with axis-specific weights for prospective stratification. For causal inference in real-world cohorts, propensity-score methods, including matching, stratification, and inverse probability of treatment weighting (IPTW), may be combined with doubly robust estimators or targeted maximum likelihood estimation (TMLE) to estimate average treatment effects and prespecified subgroup effects. Causal forests and uplift models may estimate conditional average treatment effects (CATEs) to identify candidate high-response subgroups for future enrollment or protocol testing; these estimates should not be interpreted as validated individual treatment effects or used directly for clinical titration without prospective validation. Hierarchical Bayesian models with partial pooling may support both tracks by stabilizing multicenter estimates while preserving and clearly communicating uncertainty [35–116].

### Composite Response Index (CRI)

The CRI must be effective, causally aware, and regulator-ready. Candidates are direction-harmonized and Z-standardized; baseline weights are set by a data-driven and expert-prior hybrid using three screens: effect size and stability, correlation with functional endpoints, and cross-center measurability, then locked. MCID is estimated in pilots (approximately 0.3–0.5 SD), with guardrails on weight drift during external validation. Multiplicity is controlled (e.g., Hochberg or Benjamini–Hochberg), and robust losses (Huber or quantile) protect against extremes. For prospective studies and regulatory submissions, the CRI formula, weights, missing-data rules, and thresholds should be preregistered and version-locked, with change-control SOPs, reproducible computational environments, and analysis scripts provided [104, 114].

### Digital twin, validation, monitoring, and ethics

A lightweight “digital twin” could support the predict → titrate → verify process as an in-treatment monitoring and control framework. State-space models, Kalman filtering, Bayesian dynamic linear models, and, where appropriate, modern deep time-series models may integrate wearable data, including HRV, sleep, and steps, with A-layer laboratory measurements to update a subject-specific state vector. Dose inputs, including pressure, duration, frequency, air breaks, and PTI, may serve as control variables. MPC could propose candidate micro-adjustments within protocol-defined efficacy–safety boundaries and generate two classes of alerts: insufficient response, such as plateauing improvement, and safety-boundary alerts, such as an increase in oxidative-stress markers or the emergence of clinical prodromes. These alerts could be linked to candidate “micro-titration cards,” such as a 0.2-ATA pressure reduction, segmented exposure, or a longer recovery period.

Local and global explanation methods, including SHapley Additive exPlanations (SHAP), may indicate whether mitochondrial, endothelial, or inflammatory signals contribute to a recommendation. Because model outputs may be sensitive to assay platform, center effects, and missing-data patterns, CRI-related features should be constructed using prespecified variable definitions, harmonized units, platform-specific calibration, and version-locked preprocessing rules. When laboratory-developed tests (LDTs), in vitro diagnostic (IVD) assays, or research-use-only platforms are mixed across centers, raw biomarker values should not be pooled directly; instead, bridging studies, center-calibrated Z-scores, batch-level quality control, and platform-stratified sensitivity analyses should be used before biomarkers are entered into CRI or model-prediction pipelines.

Model development should use nested cross-validation for internal validation, rolling-origin splits for temporal validation, and external multicenter validation. Model evaluation should report discrimination and calibration using the area under the receiver operating characteristic curve (AUC), area under the precision–recall curve (AUPRC), calibration plots, and Brier score for classifiers, and R², mean absolute error (MAE), and distributional alignment for continuous outcomes. Candidate thresholds should be accompanied by decision-curve analysis to quantify potential net benefit. Following deployment, programs should monitor distribution shift, performance drift, algorithmic bias, and data quality through predefined alerting and rollback procedures; all updates should undergo change control and performance-equivalence testing, with historical versions retained. Bias and drift monitoring should be stratified by center, indication, phenotype, sex, age group, comorbidity burden, assay platform, and chamber protocol whenever sample size allows. Intended use, inputs and outputs, candidate thresholds, operational limits, and human oversight should be documented. De-identification, data minimization, access auditing, and, when data cannot leave participating sites, federated or secure multiparty learning should be implemented. If algorithms influence dosing recommendations or automated decision gates, Software as a Medical Device (SaMD) and Artificial Intelligence–enabled Medical Device (AI-MD) documentation, including risk management, lifecycle management, usability, clinical-performance equivalence, and change-impact assessment, should be maintained [22, 23, 108, 110].

Near-term deployment should be conservative. Before any autonomous or semi-autonomous dose recommendation, models should first be used as clinician-facing dashboards that flag missing data, candidate safety-threshold exceedances, and protocol deviations. Prospective silent-mode validation, predefined model-freeze periods, bias monitoring across centers and phenotypes, documented human-override procedures, and audit trails should be required before MPC outputs are permitted to influence dosing. Therefore, digital-twin and MPC outputs should be interpreted as decision-support signals for prospective validation rather than autonomous clinical dose-adjustment instructions.

## Quality, compliance and safety

### Laboratory-developed test/in vitro diagnostic (LDT/IVD)

Precision HBOT depends on biomarkers that perform consistently across sites. Laboratories should implement a comprehensive quality system that includes method validation, routine quality control, and external comparison. Under LDT/IVD pathways, validate accuracy, precision, linear range, limit of detection/limit of quantification (LoD/LoQ), matrix effects, carryover, and stability, and document these in SOPs covering pre-analytical (sampling, transport, storage), analytical (platforms, reagent lots), and post-analytical steps (normalization, unit conversion, reference intervals) [23, 80, 89, 111]. Daily operations should include within- and between-run quality control with prespecified decision limits and out-of-control procedures (e.g., Westgard rules), as well as instrument calibration and maintenance logs. Participation in external quality assessment/proficiency testing (EQA/PT) is routine, not optional [23, 109, 111]. Biomarkers used in CRI construction should be mapped to their analytical status, including LDTs, IVD assays, research-use-only assays, and functional or device-derived measurements. For cross-center programs, conduct method equivalence studies and mixed-effects bias estimation, agree on conversion or equivalence formulas, and version-lock them to prevent platform or reagent changes from introducing undetected drift [23]. When LDT and IVD platforms are mixed, raw biomarker values should not be pooled directly; instead, bridging studies, systematic-bias estimation, version-locked conversion equations, platform-specific Z-score transformations, or platform-stratified CRI models should be used. Batch-level quality control, inter-laboratory exchange samples, predefined recalibration triggers, and documentation of platform, reagent-lot, operator, and software-version changes should be maintained before CRI outputs are compared across centers or used in multicenter RWE analyses. Clinical functional tests (e.g., FMD/RHI, VO₂max) require operator training and credentialing, a standardized environment (no caffeine, controlled temperature and humidity, mandated rest), and periodic blinded duplicates to assess reproducibility [54, 80].

### Data governance and privacy

Multi-source data should be integrated under a single model with a detailed data dictionary (variable names, units, codebooks, missingness conventions) and comprehensive metadata (sampling time, device/firmware, operator, lot numbers, pre-processing conditions). Time alignment to exposure windows, air-break timing, and compression or decompression rates is explicit so dosimetry and biomarkers can be interpreted together [21, 23, 110]. Access control follows irreversible de-identification and data minimization, with end-to-end audit trails. Key analyses are delivered in reproducible containers with version-controlled code [22, 110]. For cross-site collaboration, use tiered authorization or federated or pooled-parameter analysis to limit out-of-domain data movement [22]. For wearables and remote PROs, pre-specify device harmonization, adherence thresholds, and sampling cadence, and maintain a data quality dashboard for missingness, noise, and outliers [109]. Retention, backup, and destruction follow a lifecycle policy. Consent forms clearly state use, duration, and withdrawal mechanisms in plain language [21, 110].

### Clinical safety monitoring and stop-rules

Safety is integrated into the prescription process. An oxygen-toxicity checklist links symptoms with objective trends: pulmonary risk combines cough or chest tightness with pulmonary function test (PFT)/DLCO results and an oxidative-injury panel (F2-isoprostanes, 8-oxo-dG) for composite assessment [86, 89, 91]. CNS risk is initially assessed by prodromes (visual or sensory auras, nausea, agitation, perioral twitching); research programs may include EEG. Any warning sign prompts de-escalation: reduce pressure or duration, introduce or extend air breaks, add recovery days, or suspend therapy if necessary, documenting reversibility [90, 111]. Common adverse events – barotrauma or ENT issues, glycemic variability, anxiety, or insomnia – are managed proactively: equalization training and pressure-balance guidance, scheduling to avoid late-evening high intensity, and medication review for agents that increase pulmonary or CNS sensitivity [24, 111]. Trials and multicenter cohorts appoint an independent DSMB, report serious adverse events within 24–72 h with causality assessment and corrective and preventive action (CAPA), and conduct interim safety reviews at predefined thresholds [105, 111, 112].

Reversibility of oxidative-damage markers should be handled empirically rather than assumed. F2-isoprostanes and 8-oxo-dG can decline after exposure reduction or improved recovery, but the time course depends on baseline inflammation, renal clearance, tissue burden, sampling matrix, and cumulative oxygen exposure. A practical safety rule is to resume the prior protocol only after symptoms resolve and repeat markers return toward the individual baseline or stabilize within the predefined acceptable change band; otherwise, the next sessions should restart at lower PTI with longer air breaks and earlier reassessment.

More broadly, oxidative-stress dysregulation has also been reported in non-HBOT clinical and environmental contexts, including polycystic ovary syndrome and pollution-related reproductive outcomes, supporting the general biological relevance of redox imbalance while not serving as direct evidence for HBOT dosing decisions [117–121].

### Governance, reporting and continuous improvement

Transparency starts with prospective registration of trials and SAPs, including primary, secondary, and exploratory endpoints, time windows, multiplicity control, and missing data plans. RWE studies are pre-registered using a target-trial framework (eligibility, comparators, endpoints, follow-up) [35, 105, 109]. For algorithms or composite endpoints such as the CRI, provide the full specification – composition, weights, formula, MCID/thresholds, versioning – along with change-control SOPs and rollback plans [104]. Define a Minimal Reportable Biomarker Set (MRBS): at least one to two core markers per A/B/C layer, unified units and reference intervals, QC status displayed, and figures annotated with sampling windows and relative dose (PTI/cumulative exposure); note any external validation or cross-checks [20]. Reporting follows CONSORT/CONSORT-AI, STROBE, TRIPOD/TRIPOD-AI, and CHEERS as applicable, with an open data dictionary and key analysis scripts or pseudocode to enable verification [20, 104, 105, 109]. Quality is maintained by the “people–machine–environment” triangle. Build a competency matrix and provide continuing training (sampling, FMD/RHI operation, flow cytometry gating, MS platforms, privacy, and data entry), maintain skills with blinded samples and re-tests, and use dual review for critical roles within a no-blame culture that surfaces risks early [23]. Equipment programs include metrological calibration, preventive maintenance, consumables logs, and environmental monitoring; firmware, software, or reagent changes trigger mini-revalidation [23]. Patient-facing materials – consent forms, risk–benefit leaflets, emergency contacts – use clear, multilingual formats to improve comprehension and adherence [21]. All of the above supports a unified risk management and improvement loop: failure mode and effects analysis (FMEA) to identify high-risk steps; key quality indicators (QC out-of-control rate, sampling bias rate, data missingness rate, serious adverse event (SAE) rate, stop-trigger rate); and monthly or quarterly reviews with closed-loop CAPA [23]. Documentation for software- or algorithm-supported decisions follows ISO 14,971 (risk), ISO 13,485/ISO 15,189 (quality/medical labs), IEC 62,304 (software lifecycle), and IEC 62,366 (usability). If tools meet SaMD/AI-MD criteria, maintain clinical performance equivalence, change impact, and post-market performance dossiers [22, 23, 110]. Finally, embed health economics and equity: collect resource use and cost data in RWE, monitor access by sex, age, and socioeconomic status (SES), and avoid sampling or device thresholds that create systematic bias, so precision HBOT remains safe, effective, and affordable [20, 21].

## Challenges and outlook

A central implementation challenge is that the full biomarker architecture is not yet feasible for all HBOT centers. Cost, sample logistics, assay turnaround time, equipment access, staff training, reimbursement, data governance, and patient burden may limit adoption. For this reason, the framework should be rolled out in tiers: a minimum reportable clinical panel for routine care, a standard panel for multicenter registries, and an enhanced panel for platform trials and mechanistic studies. This staged approach reduces over-medicalization while preserving a path toward validation.

### Harmonization and dose equivalence

The main obstacle to review-ready, multi-center precision HBOT is methodological heterogeneity. Chamber types, compression and decompression rates, air-break patterns, oxygen sources, masks, and operator routines alter the exposure waveform even when nominal pressure and time are matched, undermining biological equivalence and meta-analytic synthesis [80, 111]. A practical baseline is to report dose using two harmonized metrics: PTI and cumulative oxygen exposure, and to use A-layer proximal biomarkers as a bio-dosimeter for center-to-center calibration through cross-center regression or hierarchical Bayesian adjustment. Standard operating procedures – including compression and decompression curves, intermission timing, and in-chamber activity limits – should be version-locked, with mini re-validation after any device or process change. “Dose comparability” should be a required item in peer review and regulatory dossiers [23, 97, 111].

### Controls, blinding, and ethics

Creating a convincing sham is challenging because somatic cues, such as noise and pressure sensations, can unblind participants; however, room air or very low-pressure controls may still produce small physiological effects [32, 113]. Trials should use “experience-equivalent, effect-minimal” controls – such as mock pressurization cues, identical masks, and matched dwell times – and prospectively assess the success of blinding. Placebo and contextual effects, including participant expectation, staff interaction, chamber experience, and symptom fluctuation, should be explicitly measured or minimized through blinded outcome assessment, credibility questionnaires, and sensitivity analyses. When full blinding is not feasible, prioritize objective endpoints, operator blinding, independent adjudication, and sensitivity analyses. Ethically, apply a “minimal risk and fair access to benefit” approach, using crossover or delayed-treatment designs, prespecified rescue or stop rules, and post-trial access for likely beneficiaries [21, 105, 109].

### Long-term outcomes and value

Short-term A-layer gains do not necessarily lead to reduced hospitalization, disability, mortality, or sustained improvements in quality of life and function. Real-world studies should use target-trial emulation with preregistered populations, comparators, time windows, and endpoints, while health-economic evaluations (cost-effectiveness, utility, budget impact) and resource-use tracking (visits, inpatient days, wound-healing time, return to work) are conducted in parallel [21]. In neuro-rehabilitation, chronic wounds, and cardio-metabolic risk, linked registries that connect early CRI gains to mid-term function and long-term hard outcomes provide a pragmatic “last-mile” pathway [20, 21, 35–85].

### Co-therapies and heterogeneity

Exercise, nutritional timing (e.g., ω-3 and protein), red/near-IR light, and drugs (GLP-1RA, SGLT2i, statins/PCSK9i) may synergize with or interfere with HBOT along mitochondria–endothelium–inflammation axes [75, 76, 99]. These interactions are best tested in platform trials using early A-layer gates to determine whether a combination merits continuation. Hierarchical Bayesian models with interaction terms, along with CATE/uplift estimation, can identify which phenotype × combination pairs yield the greatest benefit, shifting co-therapy from empirical stacking to strategy-based pairing [103, 104].

### Translation and outlook

Regulatory and payment pathways will increasingly favor a “diagnostics (composite biomarkers) plus dosing strategy” bundle rather than a generic “device plus procedure.” Submissions should specify biomarker performance and reproducibility, auditable titration rules, and software versioning and change control. Where SaMD or AI-MD applies, include risk management, lifecycle, and usability documentation. Multi-center external validation and real-world evidence can support staged access and payment pilots in defined populations, while a minimal reportable biomarker set and clear stop rules lower adoption barriers for hospitals and payers [20–22, 110]. Cross-cutting priorities are: (i) monitoring and recalibration for data and model drift as platforms and populations evolve, ideally with federated or pooled learning for in-domain updates; (ii) prospectively defined fairness metrics and subgroup analyses to avoid inequitable benefit; (iii) sustained operator training for FMD/RHI and flow cytometry gating; and (iv) transparent decision support that states inputs, outputs, and uncertainty to strengthen patient partnership [44, 107–109]. Looking ahead, a workable progression is: mechanism-proven → biomarkers-measurable → dose-tunable → endpoints-translatable → value-payable. Concretely, this means building a global PTI and cumulative-exposure concordance table and open data dictionary; running a platform trial and target-trial real-world evidence dual track in wounds, neuro-rehabilitation, and cardiometabolic risk; deploying clinic-ready A-layer “quick panels” and simplified functional tests to extend reach; and embedding digital-twin micro-titration so the measure–treat–evaluate loop closes in real time. With aligned standards, evidence, technology, and payment, HBOT can move from experience-based practice to a strategic, auditable medical technology [21, 97, 108].

## Conclusion

Precision HBOT treats oxygen as a measurable therapy rather than a simple setting. By integrating a three-layer biomarker architecture – Layer A for proximal, titratable biology; Layer B for pathway mediation; and Layer C for integrated, patient-relevant outcomes – with a unified dosing language (PTI and cumulative oxygen exposure), prescriptions may shift from experience-driven practice toward candidate threshold-informed, auditable protocols. A Composite Response Index, embedded in standardized sampling, quality control, and data governance workflows, is intended to support individual predictability, cross-center reproducibility, and regulatory clarity. On the evidence and translation path, platform trials with adaptive randomization may help identify effective dose–combination bundles, while target-trial–oriented real-world evidence may improve generalizability and long-term outcomes. Modern analytics, including hierarchical Bayesian models, causal inference, and digital-twin or model predictive control (MPC) approaches, may link biomarkers to dose and function in an interpretable manner, with quantified uncertainty. A parallel compliance stack, including LDT/IVD validation, EQA/PT, data privacy, and SaMD/AI-MD documentation where applicable, should support end-to-end traceability.

The near-term agenda is practical: finalize dose-equivalence mapping, establish a minimal reportable biomarker set, enforce stop rules, and conduct platform and real-world evidence programs in neurological rehabilitation, cardiometabolic risk, chronic wounds, and healthy aging, alongside health-economic evaluation, implementation studies, and reimbursement feasibility assessment. This closes the loop from mechanism to value, enabling precision HBOT to function as a strategic, reviewable medical technology. Importantly, the CRI, PTI-based dose mapping, biomarker thresholds, and titration rules proposed here remain framework-level constructs rather than validated clinical decision limits and require prospective validation before routine clinical use.

## Supplementary Information

Below is the link to the electronic supplementary material.

Supplementary Material 1

Supplementary Material 2

## Data Availability

Not applicable, as no datasets were generated or analyzed in this study.
