# Supplementary material for: Precision hyperbaric oxygen therapy: a biomarker-driven framework
Source: J Transl Med. 2026 Jul 30;24:1351. doi: 10.1186/s12967-026-08722-w (PMC13711866; doi:10.1186/s12967-026-08722-w)
Supplement: Supplementary file 1 [file 12967_2026_8722_MOESM1_ESM.docx]

Supplementary Table S1. Proposed biomarker stratification, sampling windows, and implementation roles in precision hyperbaric oxygen therapy

Supporting information for: Precision Hyperbaric Oxygen Therapy: A Biomarker-Driven Framework

| **Layer** | **Axis / pathway** | **Representative biomarkers / measures** | **Proposed sampling windows** | **Proposed role in the framework** | **Current validation status, evidence, and practicality** | **Illustrative research signals and cautions** |
| --- | --- | --- | --- | --- | --- | --- |
| **A** | Redox / oxidative stress | GSH/GSSG; F2-isoprostanes; 8-oxo-dG | Blood and/or urine; baseline; week 2 optional; weeks 4–6; weeks 8–12 | Proximal response and safety monitoring; identification of possible oxidative overshoot | Moderate translational evidence for oxidative-stress monitoring, but HBOT-specific dose thresholds are not validated. Urine or plasma assays are feasible; LC–MS/MS is preferred where available. Collection timing and pre-analytics should be standardized. | Prioritize within-person change. A sustained approximately 20–30% rise in damage indices, particularly with symptoms or falling GSH/GSSG, is an illustrative candidate trigger for review, not an automatic diagnosis or validated clinical stop rule. |
| **A** | Mitochondrial function | PBMC or platelet oxygen-consumption rate (OCR); mtDNA copy number; lactate/pyruvate ratio (L/P); ATP/ADP where feasible | Blood; baseline; week 2 optional; weeks 4–6; weeks 8–12 | Candidate assessment of energetic engagement, response prediction, and protocol-level dose optimization | Research-grade for HBOT titration. OCR is platform- and handling-sensitive and usually requires fresh cells and specialized equipment; L/P is more practical but less specific. | Interpret trajectories rather than isolated values. An OCR increase of approximately 10–15% accompanied by a decreasing L/P ratio is an illustrative pilot response pattern, not a validated clinical threshold. |
| **A** | Endothelium / microcirculation | NOx; VEGF; VCAM-1 and ICAM-1; flow-mediated dilation (FMD); reactive hyperemia index (RHI); selected perfusion imaging | Blood and functional testing; baseline; week 2 optional; weeks 4–6; weeks 8–12 | Assessment of endothelial and microcirculatory pathway engagement and candidate efficacy thresholds | FMD and RHI have established methodological guidance, but HBOT-specific decision thresholds require validation. FMD requires trained operators and controlled conditions; RHI is easier to deploy but device-dependent. | A FMD increase of approximately 1–2 percentage points or an RHI increase of approximately 0.1–0.2 may be used as an illustrative protocol-defined target. Interpret together with NOx, adhesion molecules, baseline status, and center quality control. |
| **A** | Extracellular matrix (ECM) / repair | MMP-2/9; TIMP-1; MMP-9/TIMP-1 ratio; P3NP; hyaluronan distribution; standardized wound imaging | Blood and/or imaging; baseline; weeks 4–6; weeks 8–12; longer follow-up when clinically relevant | Phenotype-specific monitoring of tissue repair, matrix remodeling, and wound trajectory | Most relevant to wound and radiation-injury phenotypes. General HBOT thresholds remain immature. Biomarker assays should be interpreted together with standardized wound photographs, area or volume measurements, and perfusion or thermal imaging when available. | Use directionality and concordance with the wound trajectory rather than universal cutoffs. Persistent MMP-9/TIMP-1 dysregulation or a stalled wound trajectory should prompt evaluation of infection, perfusion, debridement, and co-interventions before increasing oxygen exposure. |
| **B** | Inflammation / SASP | IL-6; TNF-α; CRP; selected multiplex cytokine or SASP panels | Blood; baseline; week 2–3 optional for high-inflammation phenotypes; weeks 4–6; weeks 8–12 | Verification of anti-inflammatory pathway engagement and identification of possible mediators or amplifiers of response | Common inflammatory markers are clinically established, whereas composite SASP interpretation remains context-dependent. Immunoassays are feasible; multiplex panels require batch quality control and prespecified analysis plans. | A decline of approximately 20–30% in selected inflammatory markers may be evaluated as an illustrative pilot signal. Persistent elevation should trigger assessment of infection, sleep disturbance, comorbidity, co-interventions, or possible overtreatment rather than automatic HBOT escalation. |
| **B** | Immune phenotype | Flow cytometry of T-, B-, and NK-cell subsets, including Treg and Th1/Th2/Th17 patterns; NLR where appropriate | Blood; baseline; weeks 4–6; weeks 8–12; earlier testing only in selected research cohorts | Mechanistic stratification, pathway verification, and research guidance for adjunctive interventions | Mechanistic and adjunct-guidance value is greater than routine titration value. Flow cytometry requires trained laboratories, fresh or appropriately stabilized samples, and harmonized gating strategies. | Use in complex or research cohorts. Do not change pressure or duration solely on the basis of immune-cell subsets. Persistent Th17 skew, impaired Treg balance, or related inflammatory signals should be interpreted with clinical context. |
| **B** | Cellular senescence / aging-related signatures | p16INK4a; p21/CDKN1A; selected SASP composites; exosomal or cell-associated senescence markers | Blood, PBMCs, or other validated matrices; baseline; weeks 8–12; months 3–6 for longer courses or healthy-aging research | Mechanistic stratification and assessment of aging-related pathway responses | Research-grade. Assay methods, cell-type composition, and sample handling strongly affect interpretation. These measures are not suitable as stand-alone dose-titration sensors. | Interpret longitudinally and in conjunction with inflammation, function, and clinical context. Changes should not be described as clinical rejuvenation without prospective validation and patient-centered outcome concordance. |
| **C** | Epigenetics | DNA methylation clocks and age-acceleration measures, including GrimAge, PhenoAge, and other platform-specific validated or research clocks | Buccal swab and/or blood; baseline; weeks 8–12 where justified; months 3–6 or longer for durability | Long-term integrative assessment of biological-age estimates and aging trajectories | Long-term research or registry endpoint rather than an acute dose sensor. Commercial and research assays are available, but tissue choice, platform, preprocessing, and clock selection materially affect results. | Avoid interpreting short-term shifts as clinical rejuvenation. Prespecify tissue, platform, clock, preprocessing, batch correction, and analysis strategy. |
| **C** | Proteomics / metabolomics / lipidomics | Targeted multi-protein scores; selected metabolites; lipid fractions and pathway-specific panels | Blood; baseline; weeks 8–12; months 3–6 where relevant | Integrative pathway assessment, risk stratification, and exploratory mechanism-to-outcome mapping | Exploratory-to-translational depending on platform and marker set. Targeted panels are generally more feasible and reproducible than discovery omics. Batch effects and multiplicity require prespecified quality control and statistical analysis. | Prespecify marker sets, directions, and false-discovery-rate rules. Avoid post hoc clinical interpretation, treatment claims, or dose changes based on unvalidated multi-omics signatures. |
| **C** | Microbiome | 16S rRNA sequencing; metagenomics; selected functional pathways such as short-chain-fatty-acid capacity, barrier-related features, LPS, and TMAO pathways | Stool; baseline; weeks 8–12; months 3–6 where durability is assessed | Exploratory profiling of gut–immune–metabolic and gut–brain modifiers | Exploratory. 16S sequencing is less costly; metagenomics provides greater functional resolution but requires more complex analysis. Diet, antibiotics, probiotics, bowel preparation, and collection logistics are major confounders. | Use for stratification or mechanistic interpretation rather than direct pressure, duration, or frequency changes. Prespecify collection procedures and relevant dietary and medication metadata. |
| **C** | Functional and digital phenotypes | VO2max; HRV; sleep architecture; 6-minute walk distance; grip strength; standardized cognitive composites; wound-healing metrics; validated wearable measures | Wearables and functional testing; per protocol, with key assessments at baseline, weeks 8–12, and months 3–6 | Patient-centered and functional outcome assessment; evaluation of durability and clinical relevance | Feasible for longitudinal monitoring, although device accuracy, firmware, sampling frequency, and scoring algorithms vary. Standardized devices, protocols, adherence thresholds, and metadata are required. | Use prespecified minimal clinically important differences where available. Separate efficacy from recovery burden, training effects, missing wear time, and device or algorithm changes. |
| **C** | Psychosocial and patient-reported outcomes | PHQ-9; GAD-7; fatigue, pain, symptom burden, treatment tolerance, quality-of-life, loneliness, and social-support measures | Validated scales; baseline; protocol-defined interim assessments; weeks 8–12; months 3–6 | Assessment of patient-reported benefit, adherence, tolerability, mood, and integrated health impact | Low-cost and patient-centered, but interpretation requires validated instruments, appropriate language versions, prespecified scoring, and clinical context. | Interpret with clinical interview where indicated. Worsening mood, anxiety, sleep, fatigue, or arousal may indicate poor tolerability or a need to review scheduling, intensity, recovery, and comorbid conditions. |

**Notes.**

Layer A comprises proximal candidate response and safety sensors; Layer B comprises pathway mediators and amplifiers; and Layer C comprises integrative downstream and patient-centered endpoints. A-layer markers are prioritized during the first 4–6 weeks, B-layer markers at intermediate checkpoints, and C-layer endpoints at predefined longer-term intervals.

Core assessment windows are baseline, week 2 when clinically or scientifically justified, weeks 4–6, weeks 8–12, and months 3–6. Not all biomarkers are required at every time point; marker-specific windows should reflect biological kinetics, clinical purpose, assay feasibility, and study design.

All numerical values are illustrative, protocol-defined candidate research signals rather than validated HBOT clinical decision thresholds. Interpretation should prioritize within-person change, assay and center calibration, standardized pre-analytics, clinical tolerance, and indication-specific standards.

The table should be interpreted together with the evidence and citations presented in the main manuscript.

**Proposed CRI construction.**

The Composite Response Index (CRI) may be specified prospectively as a weighted sum of directionally harmonized, center-calibrated standardized values: CRI = Σ(wiZi), where improvement is coded as positive and Σwi = 1. Initial candidate layer-level weights may be assigned within the ranges A: 40–50%, B: 25–35%, and C: 20–30%. Component selection, direction, weights, minimum clinically important difference, missing-data rules, and versioning must be prespecified and prospectively validated. At early assessment windows, a provisional A+B CRI may be used, with C-layer components incorporated at later follow-up.

**Abbreviations.**

8-oxo-dG, 8-oxo-2′-deoxyguanosine; 16S rRNA, 16S ribosomal ribonucleic acid; ATP/ADP, adenosine triphosphate-to-adenosine diphosphate ratio; CRI, Composite Response Index; CRP, C-reactive protein; ECM, extracellular matrix; FMD, flow-mediated dilation; GAD-7, Generalized Anxiety Disorder-7; GSH/GSSG, reduced-to-oxidized glutathione ratio; HBOT, hyperbaric oxygen therapy; HRV, heart rate variability; ICAM-1, intercellular adhesion molecule-1; IL-6, interleukin-6; L/P, lactate-to-pyruvate ratio; LC–MS/MS, liquid chromatography–tandem mass spectrometry; LPS, lipopolysaccharide; MCID, minimum clinically important difference; MMP, matrix metalloproteinase; mtDNA, mitochondrial DNA; NK, natural killer; NLR, neutrophil-to-lymphocyte ratio; NOx, nitric oxide metabolites; OCR, oxygen consumption rate; PBMC, peripheral blood mononuclear cell; P3NP, procollagen type III N-terminal propeptide; PHQ-9, Patient Health Questionnaire-9; RHI, reactive hyperemia index; SASP, senescence-associated secretory phenotype; Th1/Th2/Th17, T helper 1/T helper 2/T helper 17; TIMP-1, tissue inhibitor of metalloproteinases-1; TMAO, trimethylamine N-oxide; TNF-α, tumor necrosis factor-α; Treg, regulatory T cell; VCAM-1, vascular cell adhesion molecule-1; VEGF, vascular endothelial growth factor; VO₂max, maximal oxygen uptake.
