# Supplementary material for: Precision hyperbaric oxygen therapy: a biomarker-driven framework
Source: J Transl Med. 2026 Jul 30;24:1351. doi: 10.1186/s12967-026-08722-w (PMC13711866; doi:10.1186/s12967-026-08722-w)
Supplement: Supplementary file 2 [file 12967_2026_8722_MOESM2_ESM.docx]

**Supplementary Table S2. Proposed dosing dimensions, safety monitoring, and candidate titration strategies in hyperbaric oxygen therapy**

*Supporting information for: Precision Hyperbaric Oxygen Therapy: A Biomarker-Driven Framework*

| **Prescription dimension** | **Level / example** | **Monitoring focus** | **Candidate titration logic** | **Minimum viable implementation** | **Enhanced / research implementation** |
| --- | --- | --- | --- | --- | --- |
| **Pressure (ATA)** | Indication- and protocol-specific. Illustrative research ranges include mHBOT at 1.3–1.5 ATA and conventional HBOT at 2.0–2.5 ATA. | Oxidative stress; endothelial response; CNS and pulmonary risk; clinical tolerance | Begin with an accepted indication-specific or study-defined protocol. Evaluate pressure changes only after review of symptoms, A-layer trends, exposure pattern, and clinical context. | Record prescribed pressure and actual chamber profile. Biomarker findings should complement, not override, contraindications, safety standards, and clinical judgment. Avoid escalation without structured symptom and response review. | Phenotype-specific pressure comparisons, PTI-response modeling, center-calibrated A-layer markers, and prospective validation of small-step pressure changes. |
| **Oxygen fraction and delivery** | Nominal and, where available, measured FiO2; mask, hood, or chamber delivery; interruptions and leakage | Actual delivered oxygen exposure; delivery consistency; interface tolerance | Interpret pressure and time together with FiO2, delivery interface, interruptions, air breaks, and device performance. | Record prescribed oxygen concentration, delivery interface, treatment interruptions, and clinically relevant leakage or fit problems. | Time-resolved FiO2 measurement, interface and device calibration, and oxygen-waveform modeling linked to biological and clinical response. |
| **Dose metrics and exposure calculation** | Nominal PTI = ATA × oxygen-exposure minutes. Cumulative oxygen exposure = Σ(PTI × FiO2), with FiO2 expressed as a fraction from 0 to 1 and summed across sessions. | Per-session and cumulative exposure; comparison across protocols, devices, and centers | Use exposure metrics descriptively and with waveform information. Do not treat equal PTI values as biologically equivalent when peak pressure, FiO2, air breaks, compression/decompression, or patient factors differ. | Calculate and record session-level PTI and cumulative oxygen exposure using a consistent formula and unit convention. | Integrate time-resolved pressure and FiO2 curves; model peak, cumulative, intermittent, and recovery components; externally calibrate device and center effects. |
| **Session duration (min)** | Commonly 60–120 minutes, according to indication and protocol | Oxidative-stress trends; symptoms; fatigue; CNS arousal; recovery | Shorten duration or segment exposure when symptoms or candidate safety signals emerge. Consider cautious extension only when response is weak and safety signals remain acceptable. | Use a fixed, indication-appropriate session length with a symptom checklist. Add air breaks or shorten exposure for intolerance or adverse signals. | Compare duration-response relationships and continuous versus segmented exposure with early A-layer kinetics and functional recovery measures. |
| **Air-break / waveform structure** | Continuous versus intermittent oxygen; illustrative 5–10-minute air breaks | ROS peaks; CNS prodromes; pulmonary symptoms; arousal and recovery burden | Add or extend air breaks when oxidative or CNS prodromal signals emerge. Reduce continuous exposure before increasing recovery burden or abandoning a potentially responsive protocol. | Use predefined air-break rules for intolerance or safety flags and document timing and duration. | Compare waveform-specific PTI and cumulative oxygen-exposure effects, A-layer kinetics, symptom trajectories, and recovery. |
| **Compression / decompression and in-chamber activity** | Center- and chamber-specific rates; standardized activity restrictions | Barotrauma and ENT symptoms; hemodynamic and autonomic response; actual exposure profile | Modify rates for pressure-equalization difficulty, anxiety, or intolerance. Keep in-chamber activity stable when comparing biological responses. | Record compression and decompression rates, pauses, aborted sessions, and in-chamber activity. | Analyze rate-related symptoms and physiology; harmonize profiles across centers and incorporate them into device/process-equivalence models. |
| **Frequency (sessions per week)** | Commonly 3–5 sessions per week, selected by indication, recovery, sleep, fatigue, and protocol | Recovery burden; HRV; sleep quality; fatigue; symptom accumulation; pathway engagement | Reduce frequency, alternate days, or add recovery days when recovery is insufficient. Maintain or cautiously intensify only when response is favorable and safety remains acceptable. | Choose frequency using indication, patient tolerance, sleep, fatigue, and operational feasibility; record missed and delayed sessions. | Adaptive schedules based on prespecified CRI trajectory, HRV and sleep recovery, inflammatory phenotype, and platform-trial rules. |
| **Total course (weeks)** | Illustrative course length 4–12 weeks, with indication-specific exceptions | CRI and functional trajectory; plateau; delayed response; cumulative toxicity; durability | Maintain or consolidate when improvement is sustained. Review, de-escalate, stop, or redesign the protocol when efficacy plateaus, safety worsens, or no clinically relevant functional gain emerges. | Predefine reviews at weeks 4–6 and 8–12. Document the rationale for continuation, consolidation, pause, or discontinuation. | Model cumulative exposure, plateau, delayed response, post-course durability, and indication-specific response windows. |
| **Sampling / review milestones** | Baseline; week 2 optional for high-risk or trial cohorts; weeks 4–6; weeks 8–12; months 3–6 for C-layer outcomes | Safety; efficacy; pathway engagement; CRI trajectory; durability | Trigger unscheduled review for safety flags, rapid deterioration, unexpected biomarker shifts, or efficacy plateau. Match testing frequency to marker kinetics and clinical purpose. | Baseline plus fixed reviews at weeks 4–6 and 8–12; add week 2 for selected high-risk cases and months 3–6 for durability or C-layer assessment. | Adaptive or high-frequency A-layer sampling during weeks 1–4, B-layer checkpoints, and prespecified C-layer follow-up in platform trials or enhanced registries. |
| **Concomitant interventions** | Exercise; nutrition; photobiomodulation; medications; wound care; sleep interventions | Synergy; confounding; safety interactions; pathway-specific bottlenecks | Align co-interventions with the targeted pathway, but avoid simultaneous uncontrolled changes that prevent causal interpretation. | Record co-interventions and keep them stable where possible. Document dose, timing, adherence, and changes. | Protocolized timing and factorial or platform evaluation of exercise, nutrition, photobiomodulation, medication, and wound-care changes. |
| **Safety triggers, de-escalation, and restart criteria** | CNS prodromes; persistent cough or chest tightness; clinically relevant pulmonary-function or DLCO decline; oxidative-damage increase with falling GSH/GSSG; severe intolerance or other adverse events | Pulmonary and CNS oxygen toxicity; reversibility; recovery; causality and recurrence | At a warning sign, stop or pause exposure and provide air/decompression as clinically indicated. Review pressure, duration, frequency, air breaks, medications, CO2 retention, and recovery. Restart only after symptoms resolve and relevant objective measures return toward individual baseline or stabilize within a prespecified acceptable range. | Use a structured symptom and adverse-event checklist with predefined hold, medical-review, and emergency criteria. Document action, resolution, and subsequent dose reduction. | Integrate symptoms, pulmonary function, DLCO, oxidative markers, CRI trajectory, causality assessment, independent safety review, and protocol-defined re-challenge rules. |
| **Dose documentation and audit metadata** | Pressure and FiO2 profiles; PTI and cumulative exposure; air-break timing; compression/decompression; device and firmware; operator; sampling timestamps; co-interventions | Reproducibility; traceability; cross-center calibration; data and algorithm version control | Do not interpret response without sufficient exposure and process metadata. Lock units, formulas, variable definitions, and algorithm versions before analysis. | Maintain a structured treatment log and adverse-event record with standardized units and timestamps. | Use an auditable data model, device identifiers, firmware/version tracking, automated quality checks, federated or multicenter calibration, and replayable analysis pipelines. |

**Notes.**

All dose ranges, biomarker triggers, and titration rules are illustrative and indication- or protocol-specific. They are proposed for prospective evaluation and should not be interpreted as validated universal clinical decision limits.

PTI and cumulative oxygen exposure are descriptive exposure metrics rather than validated biological-equivalence formulas. They should be interpreted together with FiO2, waveform, peak pressure, air-break timing, compression/decompression rates, chamber and delivery characteristics, clinical tolerance, and A-layer trends.

Biomarker-guided titration is proposed to complement, not replace, established indication-specific protocols, contraindication screening, chamber safety procedures, clinical judgment, and emergency management standards.

The table should be interpreted together with Supplementary Table S1 and the evidence and citations presented in the main manuscript.

**Abbreviations.**

ATA, atmospheres absolute; CNS, central nervous system; CRI, Composite Response Index; DLCO, diffusing capacity of the lung for carbon monoxide; ENT, ear, nose, and throat; FiO2, fraction of inspired oxygen; GSH/GSSG, reduced/oxidized glutathione ratio; HBOT, hyperbaric oxygen therapy; HRV, heart rate variability; mHBOT, mild hyperbaric oxygen therapy; PTI, pressure–time integral; ROS, reactive oxygen species.
